# Independent Wavefront Multiplexing with Metasurfaces via Non‐Injective Transformation

**DOI:** 10.1002/adma.202511823

**Published:** 2025-10-04

**Authors:** Xiao Jin, Thomas Zentgraf

**Affiliations:** ^1^ Department of Physics Paderborn University 33098 Paderborn Germany; ^2^ Institute for Photonics Quantum Systems Paderborn University 33098 Paderborn Germany

**Keywords:** wavefront multiplexing, orbital angular momentum, OAM sorter, holography, cascaded metasurface

## Abstract

Metasurface holography offers a powerful approach for manipulating wavefronts at the nano and micro scale. Extensive research has been conducted to enhance the multiplexing capacity for diverse wavefronts. However, the independence of multiplexed channels is fundamentally restricted in techniques using single‐layer metasurfaces, resulting in unavoidable crosstalk and the need for post‐filtering of the output wavefronts. Here, a universal wavefront multiplexing concept is presented based on non‐injective transformation. By employing joint optimization on two metasurfaces, different channels can be independently designed without any constraints on the output wavefronts. To validate this approach, ultra‐compact orbital angular momentum (OAM) sorters are designed. In these experiments, the output beams from different channels can be independently mapped to 2D positions with high fineness. In another application of wavefront‐multiplexed holography, 10‐channel multiplexing is experimentally achieved with minimal crosstalk and without the need for post‐processing. These results demonstrate the independence between channels enabled by the non‐injective transformation in the method. The precise wavefront control and high multiplexing capacity underscore its potential for scalable wavefront manipulation devices.

## Graphical abstract

In this work, a universal metasurface multiplexing strategy is presented based on non‐injective transformation. By jointly optimizing two metasurfaces, multiplexed channels achieve independent wavefront control, overcoming crosstalk limits of single‐layer designs. Experiments demonstrate ultra‐compact orbital angular momentum (OAM) sorters and 10‐channel holography without post‐processing. This approach enables scalable, precise, and high‐capacity wavefront manipulation for advanced photonic applications.

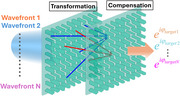

## 1 Introduction

Metasurface holography provides an approach for manipulating both the intensity and phase of wavefronts.^[^ ^1^, ^2^, ^3^, ^4^ ^]^ It has been widely applied in high‐resolution imaging,^[^ ^5^, ^6^, ^7^, ^8^ ^]^ beam shaping,^[^ ^9^, ^10^, ^11^, ^12^, ^13^ ^]^ optical information storage,^[^ ^14^, ^15^ ^]^ and 3D display.^[^ ^16^, ^17^, ^18^ ^]^ One of the central challenges in this field is enhancing the multiplexing capacity for diverse wavefronts.^[^ ^19^, ^20^ ^]^ An ideal multiplexing device should allow arbitrary mappings between input and output wavefronts, while ensuring independence among channels. However, such independence for different channels is fundamentally constrained for a single metasurface, as all output wavefronts share the same convolution kernel during the diffraction.^[^ ^21^ ^]^

**Figure 1:**
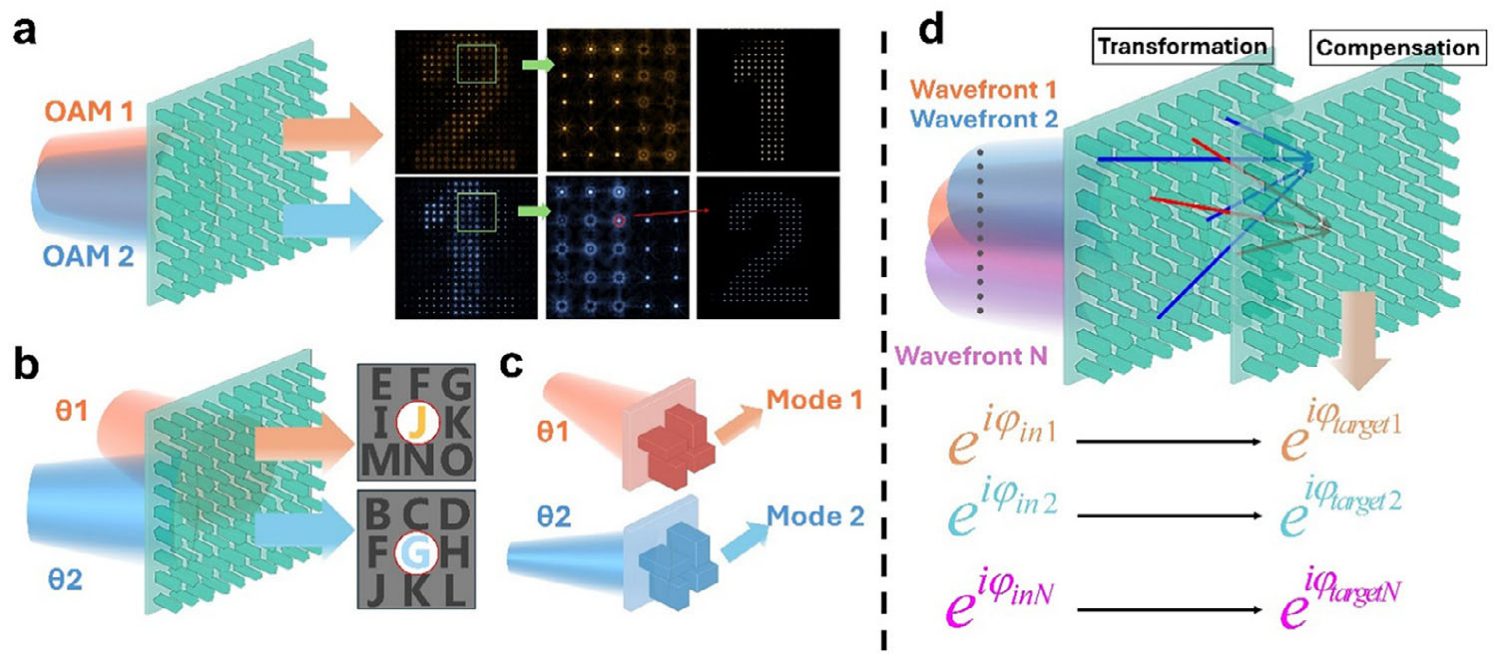
Realization of wavefront multiplexing with metasurface holograms. a) Orbital angular momentum (OAM)‐selective metasurface holography with a spatial filter array. Right: images before and after spatial filtering. b) Angle‐multiplexed metasurface holography with constrained diffraction orders. In (a,b), the output wavefronts share the same convolution kernel, preventing independent modulation. c) Angle‐multiplexed metasurface holography achieved by meta‐units with different mode responses to different angles. d) Non‐injective wavefront transformation process utilizing two cascaded metasurfaces with a transformation and a compensation plane. Below: the input wavefronts will be transferred into target output wavefronts independently.

A compromised solution to this problem is to restrict the detection regions of the output wavefronts. Techniques such as orbital angular momentum (OAM) multiplexing^[^ ^21^, ^22^, ^23^, ^24^ ^]^ and certain angle‐multiplexed holography^[^ ^25^, ^26^ ^]^ filter out the regions with unused diffraction orders (**Figure** **1**a,b). However, these methods result in a reduction in the effective imaging areas and an energy loss proportional to the number of multiplexed wavefronts.^[^ ^22^, ^27^ ^]^ Another solution involves specially designed meta‐units that support distinct modes, each responding differently to varying incident angles, thereby enabling angle multiplexing (Figure 1c).^[^ ^28^, ^29^, ^30^ ^]^ Nevertheless, as the number of multiplexed wavefronts increases, designing such meta‐units becomes increasingly complex, while the diffraction efficiency remains low.^[^ ^29^ ^]^

Cascaded metasurfaces might offer an alternative direction to address this issue. Traditionally, two‐layer metasurfaces can perform injective coordinate transformations on input wavefronts, such as log‐polar to Cartesian or spiral transformations.^[^ ^31^, ^32^, ^33^, ^34^ ^]^ These transformations can convert OAMs into independent linear phase gradient wavefronts. However, this method cannot be applied to arbitrary wavefront transformations. They must adhere to a limited set of analytical solutions dictated by the injective equations.^[^ ^32^ ^]^

Here, we propose and experimentally demonstrate a universal solution for the wavefront multiplexing problem. By employing only two metasurfaces with a non‐injective transformation, our method enables independent wavefront multiplexing without constraints on output wavefronts. The diagram of non‐injective transformation is illustrated in Figure 1d. With such a method, a single transferred point is formed by multiple initial points, and arbitrary input wavefronts can be independently mapped to target wavefronts. We experimentally validate our proposed method by designing and characterizing ultra‐compact OAM sorters, and further demonstrate OAM‐multiplexed holography without sampling constraints, which breaks the theoretical limitation of previous design strategies.

## 2 Results and Discussion

### 2.1 Independent Wavefront Mapping by Non‐Injective Transformation

**Figure 2:**
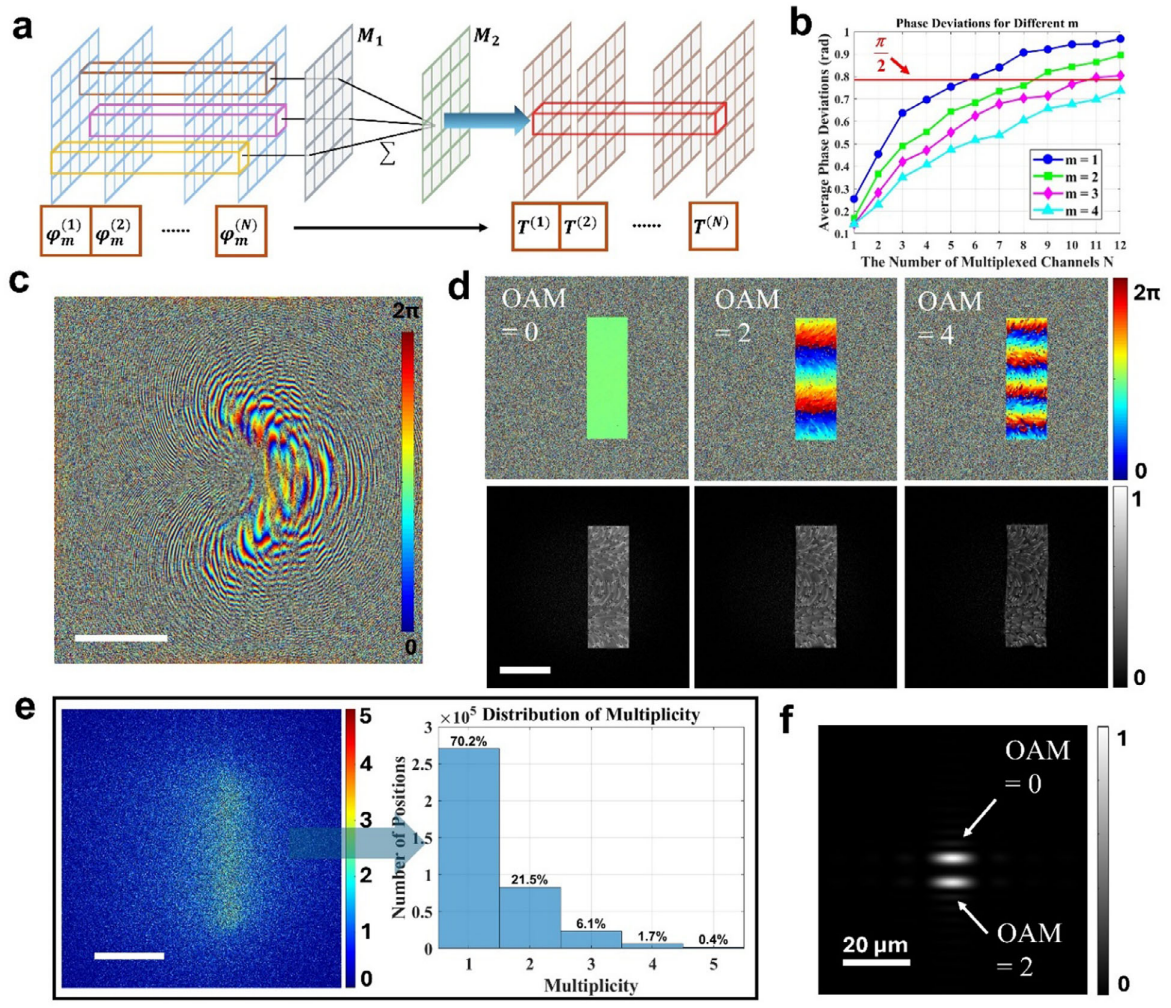
The phase mapping process and an example of an OAM sorter designed with the non‐injective transformation. a) The diagram of the phase mapping process between two cascaded metasurfaces *M*_1_ and *M*_2_. b) The average phase deviations between random target phases and optimized phases when *m* = 2. c) The phase distribution for the transformation plane of an optimized OAM sorter. d) The optimized phase (top) and intensity (bottom) distribution after the compensation plane for different incident OAMs. e) The spatial distribution and the histogram of the multiplicity on the compensation plane. f) The intensity distribution on the focal plane at a distance of 1 mm from the compensation plane for incident OAMs of 0 and 2. The scale bar in (c–e) is 100 µm.

In a conventional coordinate transformation system, an injective mapping function can be realized using two phase planes. The first transformation plane implements geometrical transformation, while the second compensation plane collimates the output beam.^[^ ^34^ ^]^ By manually selecting this transformation function, the phase corresponding to a single initial point on the transformation plane can be directed to a desired position on the compensation plane. When employing a non‐injective mapping process, as illustrated in **Figure** **2**a, the transformed point on the compensation plane becomes a linear superposition of multiple initial points (see Note S1, Supporting Information).

We start by considering a multiplexing process in which *N* input wavefronts are mapped to *N* independent target wavefronts. Thereby, the target wavefronts at a transformed point can be represented as a vector in the presence of a non‐injective mapping. If the transformed point corresponds to a multiplicity of *m*, the relationship between the input and target wavefronts can be mathematically described using a matrix equation:

(1) $$\left[\begin{array}{c} \left|E_{T^{\left(1\right)}}\right|e^{iT^{\left(1\right)}}\\ \left|E_{T^{\left(2\right)}}\right|e^{iT^{\left(2\right)}}\\ \vdots \\ \left|E_{T^{\left(N\right)}}\right|e^{iT^{\left(N\right)}} \end{array}\right]=e^{iM_{2}}\left[\begin{array}{cccc} e^{i{\varphi_{1}}^{\left(1\right)}} & e^{i{\varphi_{2}}^{\left(1\right)}} & \text{…} & e^{i{\varphi_{m}}^{\left(1\right)}}\\ e^{i{\varphi_{1}}^{\left(2\right)}} & e^{i{\varphi_{2}}^{\left(2\right)}} & \text{…} & e^{i{\varphi_{m}}^{\left(2\right)}}\\ \vdots & \vdots & & \vdots \\ e^{i{\varphi_{1}}^{\left(N\right)}} & e^{i{\varphi_{2}}^{\left(N\right)}} & \text{…} & e^{i{\varphi_{m}}^{\left(N\right)}} \end{array}\right]\left[\begin{array}{c} E_{w_{1}}\\ E_{w_{2}}\\ \vdots \\ E_{w_{m}} \end{array}\right]$$

here, $|E_{T^{\left(N\right)}}|$ and $e^{iT^{\left(N\right)}}$ denote the amplitude and phase of the *N* ^th^ target wavefront at the transformed position on the compensation plane, respectively. The expression $e^{i\varphi_{m}^{\left(N\right)}}$ represents the phase at the *m* ^th^ initial position under the *N* ^th^ input wavefront. The vector *E_w_* represents the fixed complex weight factors introduced by the transformation plane *M*_1_, which are subsequently compensated by the phase on the compensation plane *M*_2_. In the given matrix equation, the multiplicity introduces *m* free variables, while the multiplexing requirement imposes a system of *N* equations. Notably, when the input wavefronts correspond to distinct OAMs or incident angles, the resulting matrix assumes the structure of a Vandermonde matrix (see Equation S9 in Note S1, Supporting Information). When *m* ≥ *N*, the system is either underdetermined or determined. According to the full‐rank property, such a system admits a solution. Consequently, in a non‐injective transformation, independent wavefront multiplexing remains achievable.

For cases where *m < N*, the matrix equation can only yield an approximate solution with phase deviations. However, in metasurface holography, such phase deviations are acceptable since binary phase plates have already demonstrated resilience to distortions, thereby maintaining effective wavefront mapping.^[^ ^35^ ^]^ We could implement a Monte Carlo approach in a representative simulation to assess the phase deviation in the multiplexing process (see Note S2, Supporting Information). The results show an increasing trend for the achievable minimum phase deviation for larger numbers of multiplexing channels. Figure 2b illustrates this effect by showing the average phase deviation across 50 simulations for varying values of *m* and *N*. It can be observed that a larger multiplicity corresponds to a lower phase deviation. As *N* increases, the phase deviation gradually rises and eventually approaches π, indicating an ineffective matching process. We note that for an upper limit of the phase deviation of π/2 a binary phase approximation of the target phases can still be obtained. Under this condition, each channel can be regarded as independent, and holographic multiplexing can be obtained. As discussed in Note S1 (Supporting Information), Equation (1) always admits a solution when *m* = *N*. In our simulation, the number of independent multiplexing channels can reach 50 for *m* = 50. It is important to note that the number of independent multiplexing channels can be larger than *m* when the target phases exhibit an intrinsic correlation. Especially, when *m* = 1, the equation corresponds to a point‐to‐point injective transformation. According to Equation S11 (Supporting Information), the phase difference between adjacent channels for a given initial point is fixed, implying that an injective transformation cannot map an arbitrary input phase sequence to an arbitrary target phase sequence. However, the non‐injective transformation can fundamentally overcome this limitation, providing the theoretical basis for arbitrary multiplexing scenarios.

When a substantial number of transformed points are considered, a joint optimization algorithm is essential for enhancing computational efficiency and escaping local optima. For our demonstration, we introduce a practical joint optimization approach for the inverse design of the transformation and the compensation metasurfaces (see Note S3, Supporting Information). The coordinate transformation between two metasurfaces is calculated by angular spectrum theory, avoiding the inaccuracy raised by the stationary phase approximation previously used in the injective transformation design.^[^ ^33^ ^]^ To quantify our approach, we define a loss function that is the sum of three Mean Squared Errors (MSE) corresponding to the transformation efficiency, target wavefronts, and intensity uniformity of the target areas for all multiplexing channels, respectively. With this algorithm, the phase‐matching process for a small target region can be readily visualized (see Note S3, Supporting Information). As the size of the transformation metasurface increases, more initial points can be selected, enabling the target phases to be fitted with higher accuracy.

To demonstrate the potential of the non‐injective optimization, we design an OAM sorter (Figure 2c) that maps the phases of different input OAMs independently onto a rectangular area with dimensions of 80 µm × 240 µm on the compensation metasurface. Within this region, the phase along the y‐axis follows a linear phase gradient, while the intensity remains uniform. The simulation results show that the optimized output wavefronts closely match the target wavefronts (Figure 2d). For different input OAMs, the output phases are independently distributed, following the corresponding phase gradient and maintaining a uniform intensity distribution. The multiplicity of the non‐injective transformation can be analyzed by Equation S2 (see Supporting Information). Most positions with *m* > 1 are located in the target region on the compensation metasurface with 21.5% corresponding to *m* = 2 and 6.5% to *m* = 3 (Figure 2e), indicating that the non‐injective transformation contributes significantly to the mapping process. Notably, the proposed non‐injective mapping is a partial function. During optimization, the algorithm searches for suitable initial points on the transformation plane. The unmapped points that propagate outside the target area on the compensation metasurface act as background noise. For the designed OAM mode sorter, the transformation efficiency, defined as the ratio of energy transmitted from the transformation metasurface to the compensation metasurface, is 81%.

Compared to an injective transformation, the non‐injective transformation enables more precise wavefront manipulation and therefore a lower crosstalk in the final image plane. To demonstrate this improvement, we compare the results with an OAM sorter design that uses a traditional injective transformation, specifically the log‐polar transformation (see Note S4, Supporting Information).^[^ ^34^ ^]^ In the injective design, the transformation phase (Figure S4a, Supporting Information) is analytically solved with a continuous heart‐shaped phase gradient. The heart‐shaped pattern is also revealed for the non‐injective design (Figure 2c). However, the phase pattern exhibits local displacements and perturbations in multiple small regions, indicating that these phases deviate from their original positions upon mapping and overlap with others, leading to a larger multiplicity. In the injective design, most positions have a multiplicity of 1 on the compensation plane (Figure S4b, Supporting Information). Due to an insufficient number of variables to compensate for the amplitude of the weight factors, the output wavefronts have to retain a non‐uniform profile (Figure S4c, Supporting Information), which eventually results in asymmetric aberration for different OAMs at the focal plane (Figure S4d, Supporting Information). In contrast, the focal spots for the non‐injective design are smaller and symmetric in shape (e.g., see Figure 2f), making it easier to separate and discriminate different OAM values. Consequently, the non‐injective optimization algorithm achieves an improved wavefront control for every channel, resulting in an overall decrease in crosstalk and an increase in multiplexing capabilities.

### 2.2 Linear and 2D Ultra‐Compact OAM Sorters

In the following, we will design and demonstrate two ultra‐compact OAM sorters to show the wavefront control abilities of the non‐injective optimization algorithm. The traditional design strategy of OAM sorters relies on the analytical solution of injective transformations, which has several inherent theoretical limitations. On one hand, these injective transformations constrain the shape of the output wavefronts and their focused spots.^[^ ^33^ ^]^ In particular, applying a spiral transformation can improve the fineness (*F* = Δ*x*/FWHM, where Δ*x* is the spacing between adjacent sorted modes, FWHM is the full width at half maximum of the mode). However, the sorted modes become elongated, which makes them incompatible with subsequent optical processing stages.^[^ ^36^ ^]^

On the other hand, the stationary phase approximation limits the minimum size of input beams (see Note S4, Supporting Information), while the paraxial approximation restricts the minimum separation distance between two metasurfaces.^[^ ^37^ ^]^ Although some non‐paraxial solutions have been proposed, the overall device size remains at the millimeter scale.^[^ ^37^, ^38^ ^]^ However, these issues can be effectively addressed using the non‐injective optimization algorithm.

**Figure 3:**
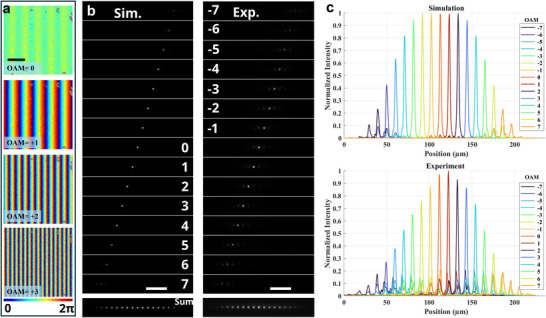
Results for a linear ultra‐compact OAM sorter. a) The optimized phase distribution in the simulation after the compensation plane for OAM = 0, 1, 2, 3. Scale bar: 100 µm. b) The intensity distributions on the focal plane for OAMs from −7 to 7 in the simulation and the experiment. Scale bar: 40 µm. c) The intensity cross‐sections along the centers of the focused points for the results in (b).

In the first design, we configure the compensation metasurface as a square region with a size of 400 µm × 400 µm. Such a configuration ensures that the focused beams maintain Gaussian intensity profiles. The target phase gradients for the horizontal wavevectors for each input OAM are set as *k_x_* = OAM × *k_0_*, where *k_0_* = 1/(12 µm). When the OAM increases by 1, the phase gradient will increase by 5.3 periods, resulting in a high fineness. Note that such target phase distributions cannot be achieved through injective transformations due to a lack of analytical solutions. **Figure** **3**a shows the optimized phase distributions after the compensation metasurface for beams with OAM ranging from 0 to 3. The optimized phase gradients match well with the target phase distributions with a transformation efficiency of 92%.

We fabricated two silicon metasurfaces on a SiO_2_ glass substrate to verify the sorter function (see Note S5, Supporting Information). At the operating wavelength, the silicon metasurfaces exhibit high efficiency.^[^ ^39^ ^]^ A lens function with a focal length of 1 mm is loaded on the compensation metasurface for the optical characterization (for details of the optical setup see Note S6, Supporting Information). In the experiment, the focused beams are shaped as small round spots and linearly sequenced along the horizontal direction with increasing OAM (Figure 3b). One can see from the intensity curves (Figure 3c) that all modes are clearly spatially separated both in simulation and experiment. The theoretical and the experimental fineness is calculated as 4.4 and 4.33, respectively. The key metrics of various OAM sorters are summarized in **Table** **1**. The experimental fineness achieved here surpasses that of most compact OAM sorters based on spiral transformations.^[^ ^36^, ^38^ ^]^ Notably, even under such high fineness, the beams remain their round small shape rather than become elongated, effectively avoiding post Gaussian‐like mode mapping.^[^ ^36^ ^]^

**Table 1:** Comparation of different OAM sorters based on coordination transformations. Device size: the size of the transformation plane and the distance between the transformation and the compensation plane).

	Output mode	Fineness	Device size	Separation efficiency
Ultracompact spiral transformation^[^ ^40^ ^]^	Elongated, linearly distributed	3.37	400 µm, 2 mm	82.6%
Generalized spiral transformation^[^ ^41^ ^]^	Elongated, linearly distributed	5.3	16 mm, 202.5 mm	Not mentioned
Non‐paraxial design^[^ ^38^ ^]^	Elongated, low aberration, linear	≈1.74	1200 µm, 11 mm	>83%
Non‐injective transformation (this work)	Gaussian shaped, low aberration, 2D distributed	4.33	400 µm, 500µm	95%

We evaluate the mode separation by using the separation efficiency defined as the ratio between the intensity of the target mode and the intensity of all the modes (Figure S8b,c, Supporting Information). For the multiplexed channels from OAM = ‐5 to 5, the average separation efficiency is 0.95 in the simulation and 0.60 in the experiment (see Note S7, Supporting Information). The decrease in separation efficiency may be attributed to imperfect fabrication of the metasurfaces and alignment errors between them. A detailed analysis of these fabrication and alignment errors is provided in Note S11 (Supporting Information). Additionally, the energy corresponding to larger OAM values decreases due to their the steeper phase gradients, which cause displacements of the propagated wavefronts on the compensation plane. This displacement leads to increased background noise and greater energy loss. Then, we test the potential of the non‐injective design by further decreasing the input beam size to 118 µm (equivalent to a smaller metasurface). The output beams show no profile difference, confirming the feasibility of a more compact device (Figure S8d, Supporting Information).

**Figure 4:**
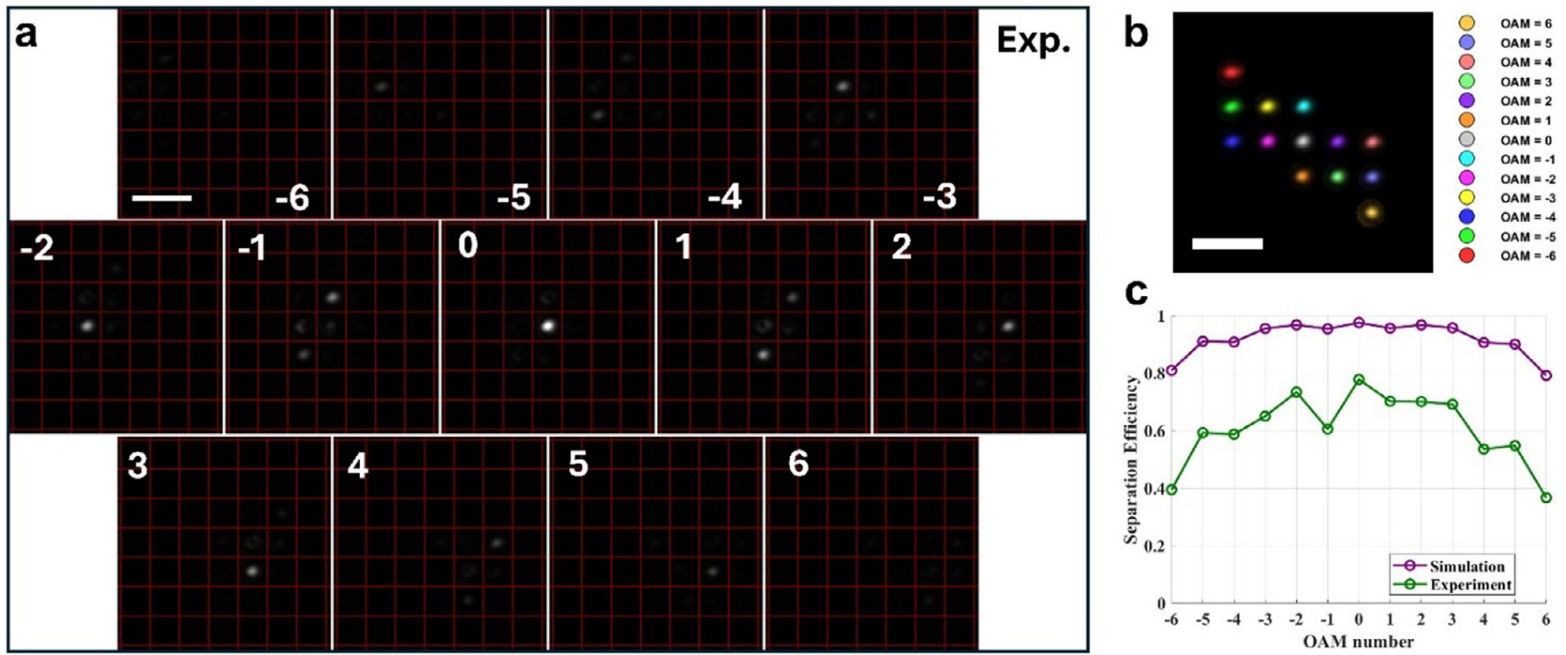
Experimental results for a 2D ultra‐compact OAM Sorter. a) Experimental intensity distribution at the focal plane for OAMs from −6 to 6. b) Superposition of all OAM modes from (a). In each channel, only the main mode is selected, normalized, and color‐coded for better visibility. c) The separation efficiency in the simulation and experiment. The scale bar in (a,b) is 20 µm.

Furthermore, by setting the phase gradient of the target wavefront to orthogonal directions on the compensation plane, we can extend the concept of the OAM sorter to two dimensions. It should be noted that a 2D sorter is mathematically impossible under an injective transformation process. For OAM = 0, ±1 and ±2, the target wavevectors are set as *k_x_* = *k_y_ =* 0, *k_y_ =* ±*k_0_* and *k_x_* = ±2*k_0_*, respectively. As expected, the focused beams are directed to different positions in the x‐y‐plane (**Figure** **4**a). We note that the unoptimized modes automatically extend to the outer positions without overlapping with other modes, eventually forming a 2D array (Figure 4b). The simulated and experimental average separation efficiencies for OAM from ‐3 to 3 are 0.96 and 0.69, respectively (Figure 4c).

Notably, the distance between the two metasurfaces (500 µm) and the size of the transformation metasurface (118 µm × 118 µm) are the smallest characteristic sizes reported yet,^[^ ^36^, ^38^, ^40^ ^]^ making the sorters ultra‐compact. Also, this device maintains the same high fineness while preserving a round beam shape without elongation. The concept of the 2D OAM sorter indicates that a non‐injective transformation not only improves the key performance metrics of OAM sorters, but also enables new sorting schemes that are theoretically forbidden under traditional injective transformation methods. Furthermore, different OAM modes can even be focused on distinct 3D positions (see Note S7, Supporting Information). These results demonstrate high spatial efficiency and hold promising applications in mode conversion to multicore fibers, fiber bundle, or waveguide coupling.^[^ ^42^, ^43^, ^44^ ^]^ When one or both of the metasurfaces are replaced with dynamically tunable materials, the system can support diverse functionalities and enable flexible channel control, offering significant advantages for next‐generation communication technologies.^[^ ^45^, ^46^, ^47^ ^]^

### 2.3 Wavefront‐Multiplexed Holography without Sampling Constraints

Naturally, the non‐injective optimization can also be applied to design multiplexed holographic phase masks. Since a holographic image can correspond to an infinite number of phase distributions, we can reduce the optimization constraints by directly replacing the target wavefronts with intensity distributions of the conjugated Fourier plane (see Method).

**Figure 5:**
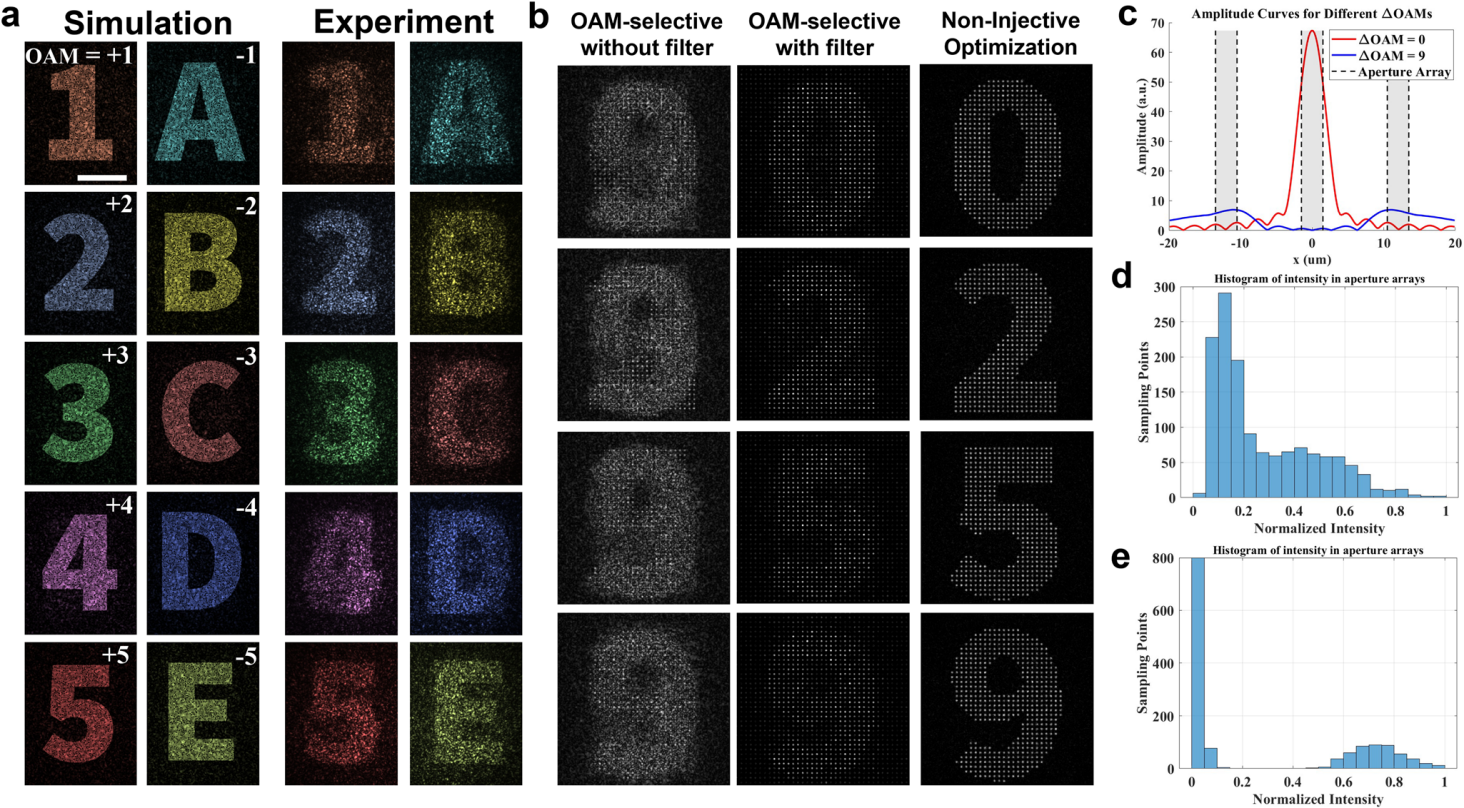
OAM‐multiplexed holography designed via a non‐injective transformation. a) The simulated and experimental result of ten independent multiplexed images corresponding to OAM input modes from −5 to −1 and +1 to +5. The images are normalized and color‐coded for better visibility. Scale bar: 100 µm. b) Comparison between results from OAM‐selective multiplexing and non‐injective optimization. The corresponding OAMs from top to bottom are 0, 2, 5, −4. c) The amplitude curves along the center of a focused point when ΔOAM = 0 and 9. The gray‐shaded areas mark the aperture arrays at the sampling positions to filter the selected OAM mode. d) The histogram of intensity for the sampling positions obtained from the OAM‐selective multiplexing method. e) The histogram of intensity for the sampling positions obtained from non‐injective optimization.

A 10‐channel OAM‐multiplexed holography is designed for OAMs from −5 to +5. Different from OAM‐selective holography where target images have to be sampled, our target images are totally continuous shapes. In the simulation, all ten channels remain clearly distinguishable with minimal noise and no noticeable crosstalk (**Figure** **5**a). Channels with positive and negative OAMs are separated into two areas for the calculation of separation efficiency. The average separation efficiency for positive and negative OAMs is 82%, which demonstrates low crosstalk. Due to imperfections in the experimental conditions, the experimental results show a larger noise (Figure 5a) with a separation efficiency of 65%.

Compared to the traditional OAM‐selective holography, our non‐injective optimization shows better energy efficiency. Here, we design two 4‐channel holograms with the same target images based on the non‐injective and OAM‐selective holography, respectively (Figure 5b). The OAM‐selective holography works only when a post‐processing aperture array filter is applied, resulting in significant energy loss. The theoretical average energy efficiency for OAM‐selective holography is limited to at most 1/*N*, which is 25% for four channels.^[^ ^18^ ^]^ Due to design imperfections, the actual energy efficiency is calculated to be 20%. In contrast, the design based on non‐injective optimization achieves an average energy efficiency of 85%, significantly surpassing the OAM‐selective holography.

Additionally, the non‐injective holography will not be limited by the resolution constraints of OAM‐selective holography, which are determined by the size of the diffraction ring generated by the largest OAM difference ΔOAM.^[^ ^27^ ^]^ If the largest diffraction ring exceeds the sampling interval, it will result in strong crosstalk among channels (Figure 5c). This crosstalk leads to a non‐uniform intensity distribution (Figure 5d), with a calculated coefficient of variation of 0.72. In contrast, the coefficient of variation for the non‐injective holography is 1.22, which represents a higher image contrast and low crosstalk among channels (Figure 5e). Importantly, the advantages of non‐injective holography are not limited to performance enhancement. The absence of a sampling requirement indicates that non‐injective holography can intrinsically overcome the theoretical constraint of channel dependence in single‐layer metasurfaces.

Beyond OAM multiplexing, the non‐injective optimization can accommodate various independent multiplexing functions. Such capability is further demonstrated with two examples in Note S9 (Supporting Information): one demonstrates angle multiplexing, enabling the overlap of OAM and transverse momentum, while the other illustrates amplitude multiplexing, mapping the clock images to their corresponding time patterns. These results indicate the power of arbitrary wavefront control. To better clarify the design flexibility of our method, a comparison with different wavefront‐multiplexing holography approaches is provided in **Table** **2**.

**Table 2:** Comparation of metrics for different wavefront multiplexing holography.

	Input wavefront type	Output restriction	Energy efficiency	Crosstalk	Capacity
Multiramp helicoconical‐OAM Multiplexing^[^ ^48^ ^]^	Multiramp helicoconical‐OAM	Large sampling interval	Decrease inversely with 𝑁	SNR: 8–12 dB	16
Spatially Structured‐Mode Multiplexing^[^ ^49^ ^]^	Hermite–Gaussian or Laguerre–Gaussian mode	Not restricted	Not mentioned	High	6
Super‐resolution OAM‐multiplexing^[^ ^27^ ^]^	OAM	50 holograms are required for analogous coherent	Decrease inversely with 𝑁	SNR: ≈15 dB (81 channels)	201
Angle‐multiplexed holography^[^ ^29^ ^]^	Tilted plane wave	Not restricted	<36%	Low	4
Non‐injective transformation (this work)	Not restricted	Not restricted	15% (50 channels)	SNR: 10.46 dB (50 channels)	More than 100

Last, we also evaluated the multiplexing capabilities of our algorithm. The proposed design strategy remains effective even when operating with up to 100 channels (see Note S10, Supporting Information). It is worth noting that our algorithm does not demand extensive computational resources and can be executed on a standard personal workstation. For example, in the simulation shown in Figure 5a, each iteration took ≈ 2.3 s, and ≈ 200 iterations were sufficient to achieve reliable results.

## 3 Conclusion

The mechanism of wavefront multiplexing is explained in the non‐injective transformation process. Building on this concept, we present a universal optimization method for independent wavefront multiplexing. In the non‐injective optimization, both the multiplexed and target wavefronts can be arbitrarily chosen.

Compared to previous wavefront multiplexing strategies, our method eliminates the limitation of low spatial efficiency caused by filtering higher diffraction orders and does not require specially designed meta‐units. Additionally, the non‐injective optimization can enable ultra‐compact designs with high energy efficiency and high‐capacity multiplexing. In our simulation, the non‐injective optimization can achieve a multiplexing capacity of up to 100 channels. Notably, this capacity could be higher with improved optimization or the use of larger metasurfaces. Moreover, the non‐injective optimization remains compatible with multiplexing techniques that leverage additional physical parameters, such as polarization^[^ ^50^, ^51^, ^52^, ^53^ ^]^ and wavelength,^[^ ^17^, ^54^, ^55^, ^56^, ^57^, ^58^ ^]^ enabling a greater overall multiplexing potential.

The non‐injective optimization strategy provides a versatile framework for addressing wavefront multiplexing challenges. It is particularly beneficial in application scenarios where compactness and high capacity are essential, such as optical communication,^[^ ^59^, ^60^, ^61^ ^]^ encryption,^[^ ^62^, ^63^, ^64^ ^]^ optical neuron network^[^ ^65^, ^66^ ^]^ and quantum devices.^[^ ^67^, ^68^ ^]^ Its flexibility and efficiency make it a promising approach for advancing next‐generation optical systems.

## 4 Experimental Section

### Configurations used in the Non‐Injective Optimization Algorithm

In the simulation, the operating wavelength was set to 785 nm, with a unit‐cell size of 400 nm × 400 nm. Unless otherwise specified, the transformation metasurface and the compensation metasurface both have a size of 400 µm × 400 µm, while a larger simulation area of 800 µm × 800 µm was used to prevent energy loss beyond the metasurfaces. The distance between the two metasurfaces is 500 µm. For the linear ultra‐compact OAM sorter, the channels with OAM values of 0, ±2, ±3, and ±4 are optimized. The imaging plane, which corresponds to the focal plane for the OAM sorter and the conjugated Fourier plane for holographic images, was located 1 mm from the compensation plane. The input wavefronts are Gaussian beams with a diameter of 370 µm. Further details regarding the non‐injective optimization algorithm can be found in Note S3 (Supporting Information).

### Design of Meta Units and Fabrication of the Metasurfaces

The silicon metasurfaces are designed and optimized using rigorous coupled‐wave analysis (RCWA). The substrate is SiO_2_ with 600 nm silicon rectangular pillars, arranged in a 400 nm period. Pillar widths and lengths are swept from 80 to 280 nm, with a 1 nm step. To reduce zero‐order diffraction from fabrication imperfections, input beams and output holographic patterns operate in orthogonal channels. The transformation plane employs Pancharatnam‐Berry phase units to convert left circularly polarized (LCP) light to right circularly polarized (RCP) light, while the compensation plane uses isotropic units for phase modulation in the RCP channel.

The fabrication process is as follows: first, a 600 nm silicon layer is deposited onto a glass substrate by plasma‐enhanced chemical vapor deposition (PECVD). The pattern was then written onto a Polymethylmethacrylate (PMMA) layer using electron beam lithography, followed by the development and deposition of a 20 nm chromium mask. Inductively coupled plasma (ICP) etching was used to form silicon pillars, and finally, the chromium mask was removed through wet etching. The detailed design of meta‐units and the fabrication process of the metasurfaces are provided in Note S5 (Supporting Information).

### Characterization Setup

The incident beam was generated by an ultra‐high‐power supercontinuum fiber laser (Fianium WhiteLase SC480) and expanded using a telescope system. OAM generation was achieved via a phase‐only spatial light modulator (Holoeye LETO‐3). The beam was modulated to LCP and slightly focused on the transformation metasurface. A 10× objective lens (NA = 0.3) was used to image the focal plane. After filtering the LCP light, the intensity distribution was recorded using a CMOS camera (IDS U3‐3680XCP‐M‐NO). Further details can be found in Note S6 (Supporting Information).

## Conflict of Interest

The authors declare no conflict of interest.

## Supporting information



Supporting Information

## Data Availability

The data that support the findings of this study are available from the corresponding author upon reasonable request.
