# Supplementary material for: Independent Wavefront Multiplexing with Metasurfaces via Non‐Injective Transformation
Source: Adv Mater. 2025 Oct 4;38(3):e11823. doi: 10.1002/adma.202511823 (PMC12801359; doi:10.1002/adma.202511823)
Supplement: Supplementary file 1 — Supporting Information [file ADMA-38-e11823-s001.docx]

**Supplementary Information for**

Independent Wavefront Multiplexing with Metasurface via Non-Injective Transformation

Xiao Jin^1,2^, Thomas Zentgraf^1,2^

1. Department of Physics, Paderborn University, Warburger Straße 100, 33098 Paderborn, Germany
2. Institute for Photonics Quantum Systems, Paderborn University, Warburger Straße 100, 33098 Paderborn, Germany

**Supplementary Note 1: Non-injective transformation process**

In an injective coordinate transformation system with a lens with a focal length of $f_{L_{1}}$ and a wave vector of *k*, the electric field propagating from the transformation plane to the compensation plane is

Here, $\varphi(x,y)$ indicates the phase at an initial position $(x,y)$ on the input wavefront. $M_{1}(x,y)$ is the phase of the transformation plane. $(x^{'},y^{'})$ represents the positions on the compensation plane. Assuming the validity of the method of stationary phase, the integration will only be contributed by all saddle points. In the case of injective transformation, only one saddle point exists, while the phase gradient of $M_{1}$ will determine the propagation directions at the point $(x,y)$. The relationship could be given by

$$\begin{matrix} \frac{\partial M_{1}(x,y)}{\partial x}=x'\frac{k}{f_{L_{1}}}, \\ \frac{\partial M_{1}(x,y)}{\partial y}=y'\frac{k}{f_{L_{1}}}\text{，} \end{matrix}$$

with

$$\begin{matrix} x'=X_{1,2}(x,y), \\ y'=Y_{1,2}(x,y). \end{matrix}$$

$X_{1,2}$ and $Y_{1,2}$ are the desired injective coordinate transformation equation. Then Equation S1 is given by

The vector $E_{w}$ denotes an extra complex weight factor introduced by the position and the phase gradient on the transformation plane. In most cases, the phase of $E_{w}$ varies faster than the input phase distribution, which leads to a phase distortion near the region of the initial points. To minimize the phase distortion, $E_{w}$ should be compensated by the compensation plane $M_{2}$:

Here, $E_{T}(x^{'},y^{'})$ denote the desired target wavefront.

If the multiplicity for the non-injective transformation, the integration of Equation S1 will become a sum of *m* saddle points, i.e., initial points. Equation S5 would also be modified as

If a N-channel wavefront multiplexing is expected, Equation S6 should be satisfied with the equation below:

To simplify the notation, *m* different coordinates will be abbreviated as the corresponding subscripts. Meanwhile, the superscript *N* denotes the *N*^th^ input and output. $|E_{T^{\left( N \right)}}|$ and $e^{iT^{(N)}}$ represent the amplitude and phase of the target wavefront at the transformed position, respectively. The relationship between the input and target wavefronts can be mathematically described using a matrix equation:

In the given matrix equation, the multiplicity introduces *m* free variables, while the multiplexing requirement imposes a system of *N* equations. In this analysis, inconsistent systems of equations are not considered, as they imply that identical input wavefronts would lead to different target functions, which contradicts the physical constraints of the system. When *m*≥*N*, the number of unknowns exceeds the number of equations, and a solution generally exists. In contrast, when *m*<*N*, an optimization algorithm is required to obtain an approximate solution that minimizes deviations. An exact solution is only possible if a subset of the equations exhibits linear dependence.

Notably, when the input wavefronts correspond to distinct OAMs or incident angles, the resulting matrix assumes the structure of a Vandermonde matrix:

According to the full-rank property, such a system admits a solution when *m*≥*N*. Consequently, in a non-injective transformation, independent target wavefront multiplexing remains achievable. To simplify the analysis, we assume the weight factors are given parameters which independent of the positions of the initial points. Although both *φ* and the weight factors are functions of the initial positions, the number of variables and unknowns does not change. Thus, the analysis of solution existence remains valid.

Specifically, for the case of *m*=1, the problem is reformulated as a phase sequencing problem for *N* phases. Assuming the target phases are randomly chosen from 0 to 2π, the total number of possible orders (permutations) is given by

$$P(N)=N!=N\times(N-1)\times(N-2)\times\cdots\times2\times1\approx\sqrt{2\pi N}\left(\frac{N}{e}\right)^{N}.$$

Meanwhile, the sequence for the transformed phase is

With $M_{2}$ and $\varphi_{1}$ varying over their respective ranges, the achievable orderings follow the variables dictated by the sum of the Euler totient function:

Clearly, as the number of multiplexed channels increases, achieving a precise correspondence between the transformed phase sequence and the randomly assigned target phases becomes increasingly challenging.

**Supplementary Note 2: Phase deviation estimation for single transformed point**


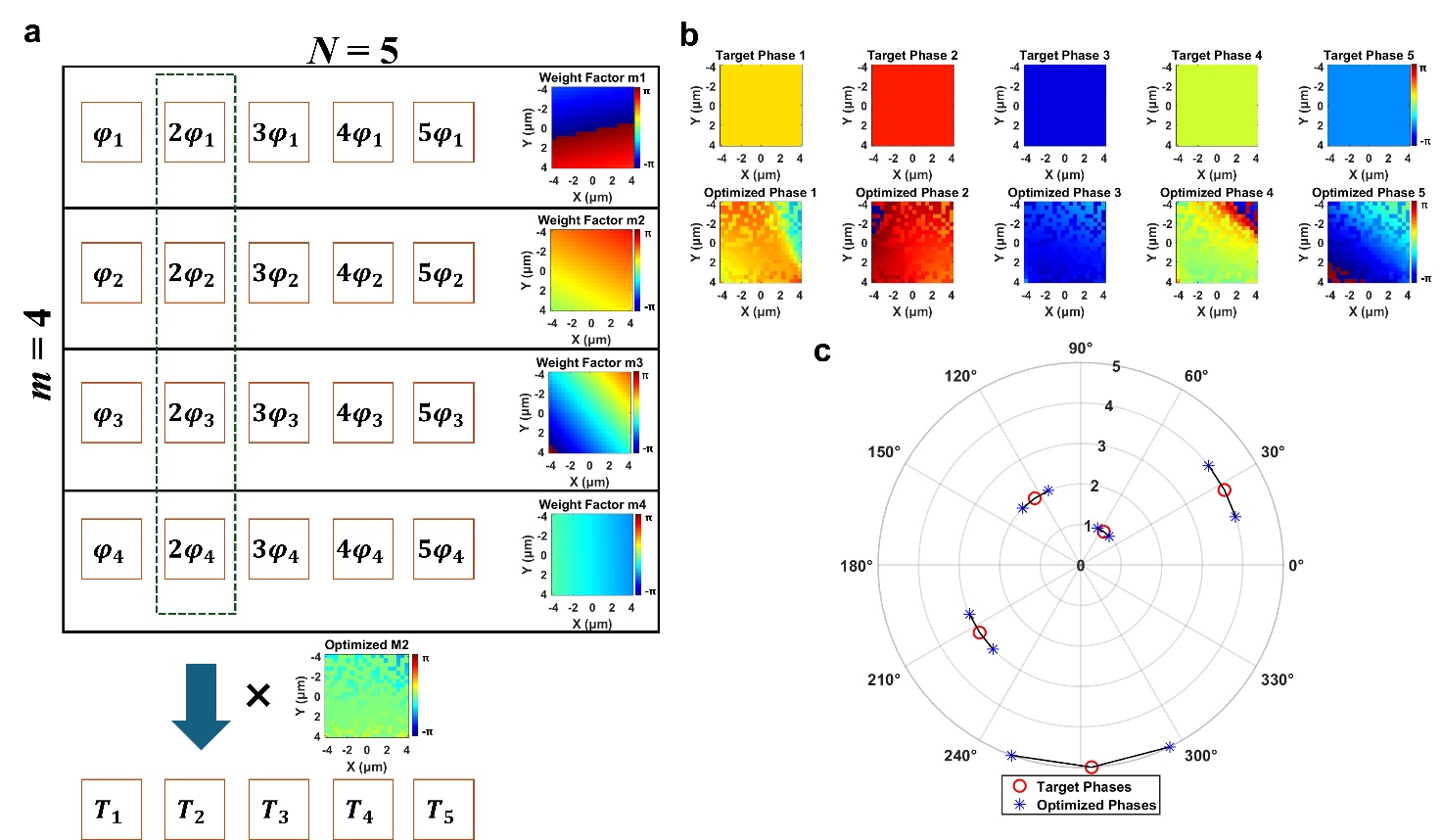


**Figure S1. The result of phase matching for *N* = 5 and *m* = 4.** **a**) The phase distributions of 4 random weight factors and the optimized phase distribution of the compensation plane in an 8 μm × 8 μm region with the pixel size of 400 nm. **b**) 5 different random target phases at a transformed point (first line) and their corresponding optimized results. **c**, The target phases and their corresponding average phase deviation in the simulation region. The radial axis of the polar plot is the number of the target phases, and the angular axis represents the phase.

An example of the multiplexing process with *N* = 5 and *m* = 4 is demonstrated within a simulation area of 8 μm×8 μm. In this simulation, weight factors and target phases are randomly assigned and fixed at a wavelength of 785 nm. Each initial point is loaded with a random spatial-variant weight factor, representing the phase gradient on the transformation plane, which is confined within the paraxial region (propagation angles < 5°).

According to Equation S9, OAMs from 1 to 5 are selected as input wavefronts. The phases of four initial points and the compensation plane are optimized to match the *N* random target phases. The optimization is performed using the Particle Swarm Optimization (PSO) algorithm with a swarm size of 400 and a maximum of 500 iterations, minimizing phase deviations. As shown in Figure S1, the target phases and optimized phases demonstrate an intensity-weighted average phase deviation of 0.45 rad in the simulated region.

**Supplementary Note 3: Joint optimization algorithm for cascaded metasurfaces and an example of phase matching process**


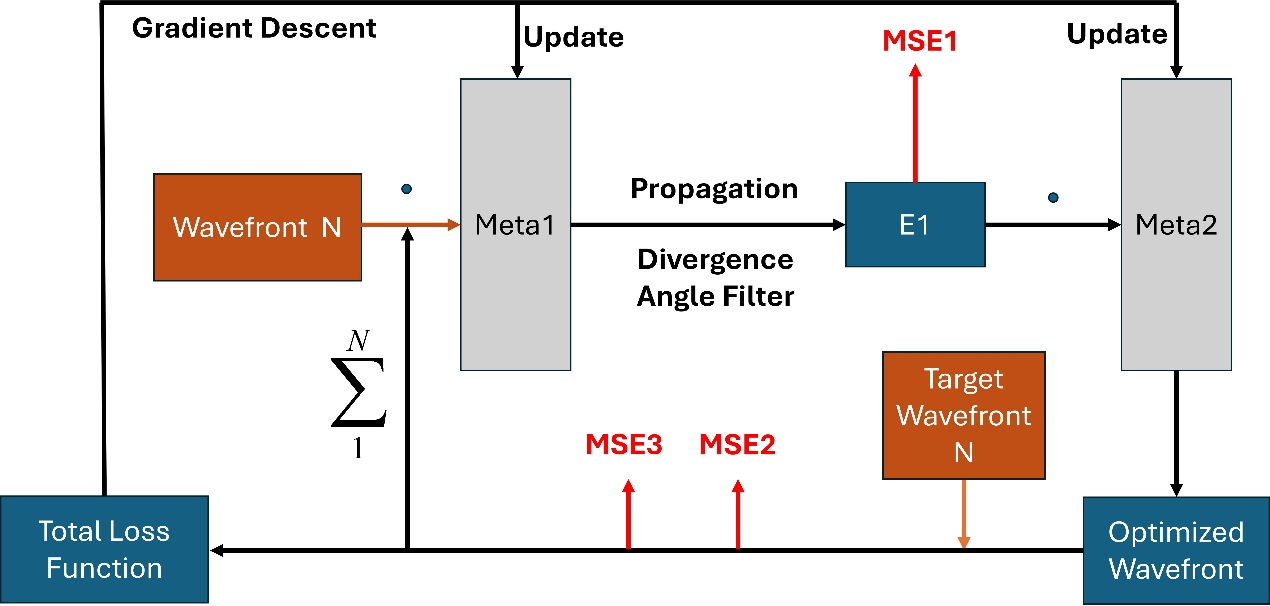


**Figure S2. The flowchart of the joint optimization algorithm for the transformation and the compensation metasurfaces.** Meta1: Transformation metasurface; Meta2: Compensation metasurface; E1: The electric field distribution before the compensation metasurface.

In this algorithm, the phase units of both the transformation and compensation metasurfaces are initially assigned random values and serve as the variables to be determined. The coordinate transformation between the two metasurfaces is performed through wave propagation. Instead of using the stationary phase approximation, we employ angular spectrum theory to achieve a more accurate solution. A divergence angle filter is applied to constrain the energy from leaking to a higher diffraction order. The transformed electric field distribution before the compensation metasurface is denoted as $E_{1}$, where MSE1 is defined as the ratio of the energy outside the compensation metasurface to the total energy.

After applying the compensation metasurface, the absolute difference between the optimized and target wavefront distributions is normalized and defined as MSE2. Additionally, the standard deviation of the intensity within the compensation metasurface region is denoted as MSE3 to ensure a uniform intensity distribution. The loss function is the sum of these three MSE values across all channels. Gradient descent is employed to minimize the loss function, with gradients computed via backpropagation. To accelerate convergence, the learning rate is halved after two consecutive increases of the loss function. The optimization process stops when the loss function reaches 5% of the starting point. The above algorithm is implemented in MATLAB.


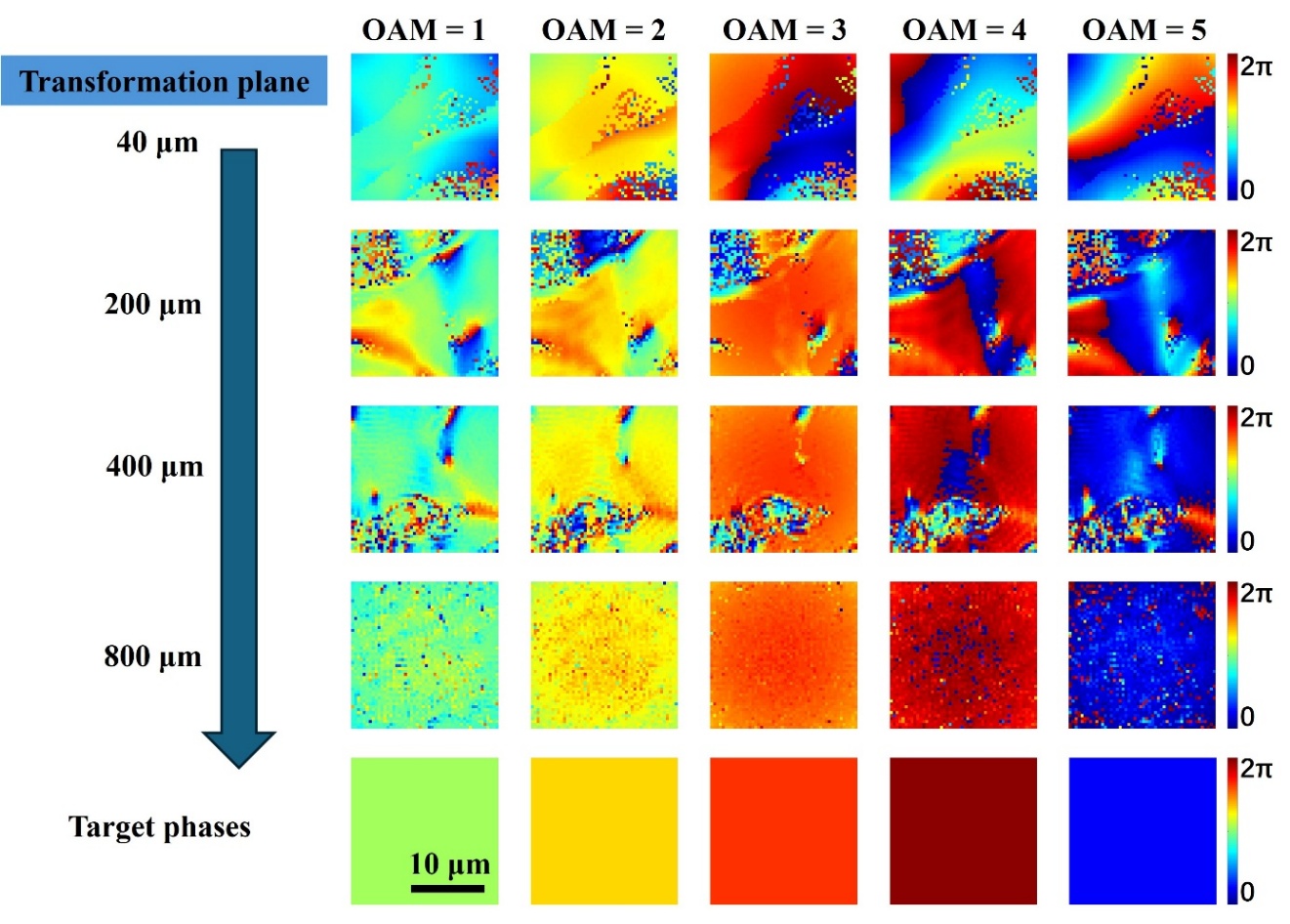


**Figure S3.** A 5-channel phase matching for a target region of 20 μm × 20 μm. From top to bottom, the size of the transformation plane increases from 40 μm to 800 μm.

Based on this algorithm, we can directly simulate the phase matching process for a small region with a size of. 20 μm × 20 μm. We present a demonstration of the five-channel phase-matching process. As shown in Figure S3, with increasing transformation plane size, the phases of the five channels progressively approach the target phases. The corresponding phase deviations for the four plane sizes are 1.107, 0.759, 0.590, and 0.345, respectively. This simulation indicates that the size of the transformation plane constrains the number of available initial points. As the transformation plane becomes larger, more suitable initial points can be identified for phase matching. However, not every region on the transformation plane is useful. Expanding the area to map the target phases inevitably leaves more regions unused, leading to reduced energy efficiency.

**Supplementary Note 4: Simulation of OAM sorters based on log-polar transformation**


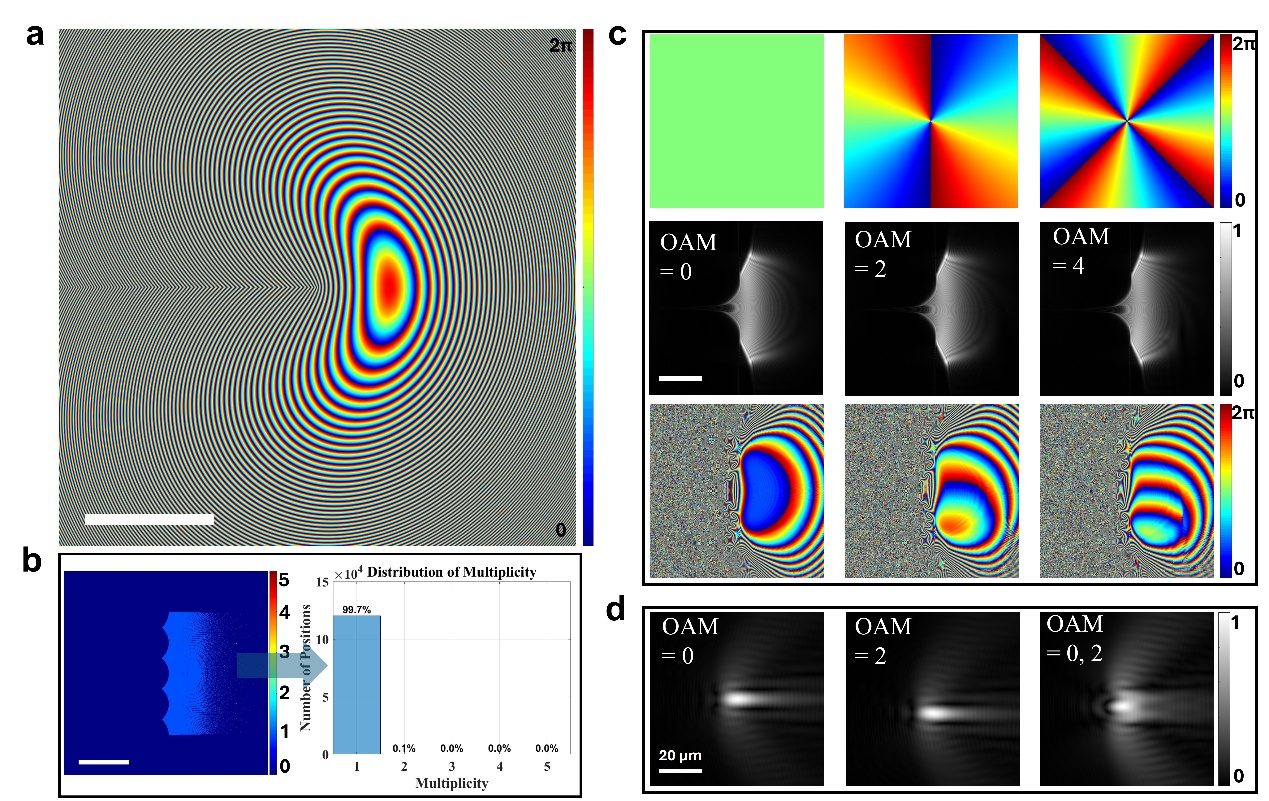


**Figure S4. Simulation results for an OAM sorter based on** **log-polar transformation. a**) The phase distribution for the transformation plane of an optimized OAM sorter. **b**) The spatial distribution and the histogram of the multiplicity on the compensation plane. **c**) The input OAMs (top), intensity (middle), and phase (bottom) distribution after the compensation plane for different incident OAMs. **d**) The intensity distribution on the focal plane at a distance of 1 mm for incident OAMs of 0 and 2. The scale bar in (**a**), (**b**), and (**c**) is 100 μm.

The log-polar transformation will conformally map log-polar coordinates in the input plane to Cartesian coordinates. The phase distributions of the transformation metasurface and the compensation metasurface are expressed as:

Here, *λ* represents the wavelength of the incoming beam, and *f* is the focal length of the Fourier-transforming lens. The parameter *a* scales the transformed image and is defined as *a* = *d*/2π, where *d* is the length of the transformed beam. Meanwhile, *b* controls the translation of the transformed image in the transverse direction and can be chosen independently of *a*. The last terms in the equations represent an extra lens function to implement a lens-free Fourier transform. To match the simulation configuration in the non-injective optimization, *d* and *b* are set as 240 μm. *f* is set as 500 μm, representing the distance between two metasurfaces.

The calculation of the multiplicity on the compensation metasurface follows Equation S2. The initial points (pixels) on the transformation metasurface will be mapped to a position $(x^{'},y^{'})$ on the compensation metasurface. At each position on the compensation metasurface, only initial points with an included angle greater than 0.1° will be considered, avoiding dense mapping from the same direction.


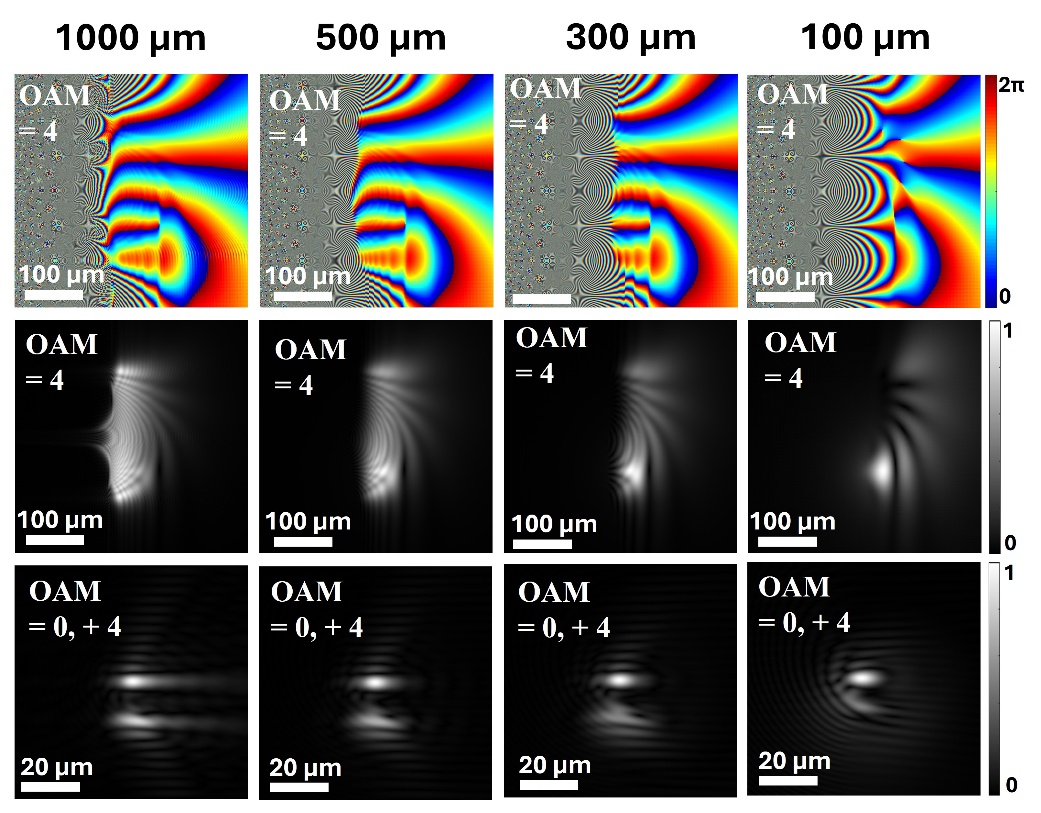


**Figure S5. The phase deviation of an OAM sorter with decreasing diameters of incident beams from 1000 μm to 100** **μm.** Top: Phase distribution after the compensation plane for input OAM = +4. Middle: Intensity distribution after the compensation plane for input OAM = +4. Bottom: Intensity distribution on the focal plane for input OAM = 0 and +4. The distance between the two metasurfaces is set to 3.5 mm.

In the central region of incident beams carrying a specific OAM, the phase exhibits rapid spatial variation, leading to the breakdown of the stationary phase approximation. As the diameter of the incident beam decreases, phase deviation becomes more pronounced. In Figure S5, we compare the phase distribution after the compensation plane for incident beams with four different diameters. When the diameter decreases to 100 μm, the focal point corresponding to OAM = +4 disappears, limiting the minimum achievable device size.

**Supplementary Note 5: Design and fabrication of the metasurfaces**


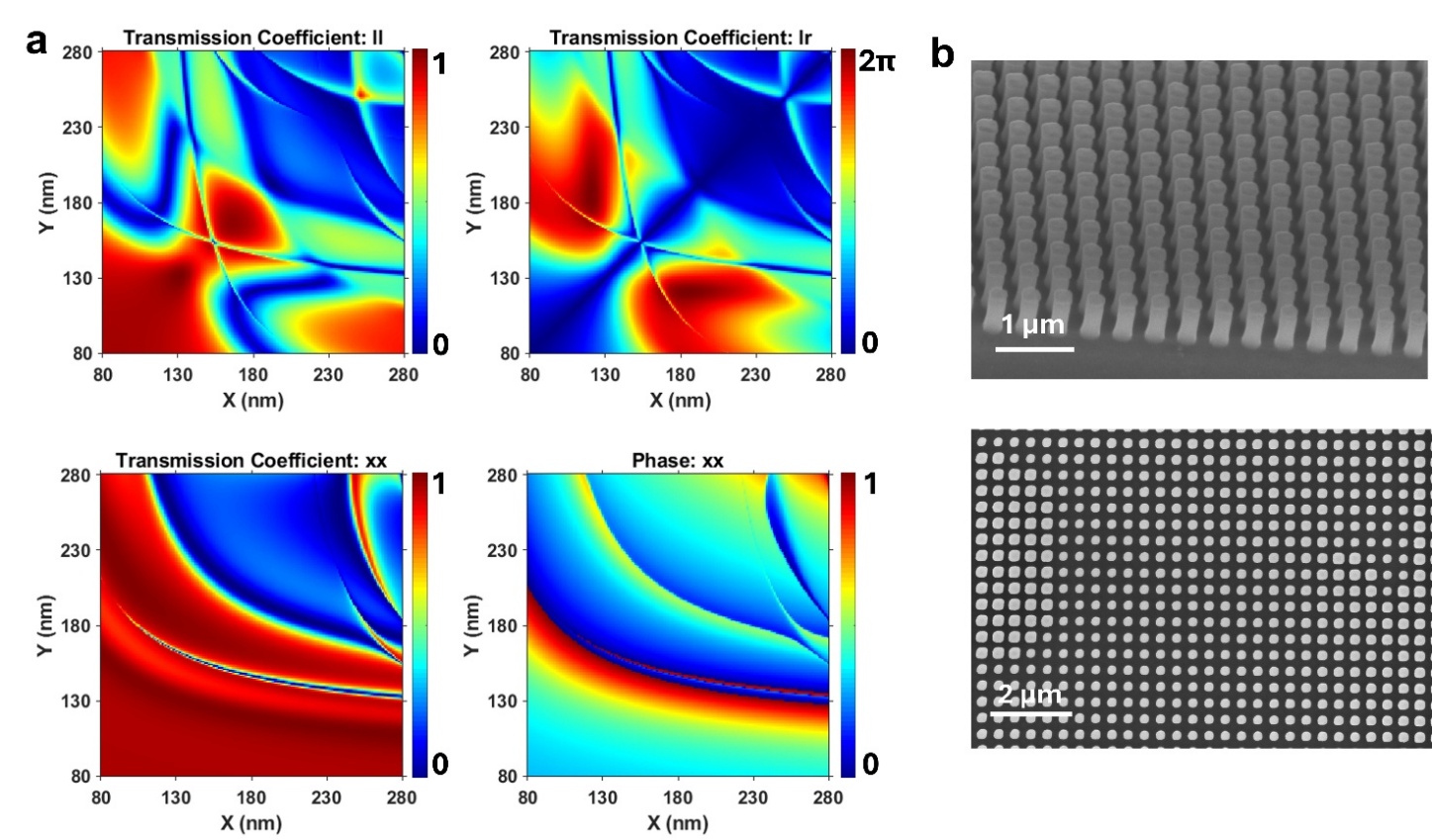


**Figure S6. Phase maps for metasurface units and the fabrication results. a**) Top: amplitude and phase maps for LCP-to-RCP conversion. Bottom: amplitude and phase maps for x-polarized light. **b**) Scanning electron microscope (SEM) images for the fabricated compensation metasurface.

Silicon metasurfaces are employed to realize the transformation and compensation planes. Rigorous coupled-wave analysis (RCWA) is utilized for the design and optimization of the phase maps. The substrate is silica, with 600 nm silicon rectangular pillars on top. The period is set to 400 nm. The width and length of the pillars are simulated in the range of 80 nm to 280 nm with a step size of 1 nm. To minimize the zero-order diffraction caused by fabrication imperfections, the input beams and output holographic patterns operate in orthogonal channels. The transformation plane utilizes Pancharatnam-Berry phase units to convert left circularly polarized (LCP) light to right circularly polarized (RCP) light. The compensation plane is composed of isotropic units, providing phase modulation in the RCP channel.

The corresponding phase maps are scanned and shown in Figure S6. For the Pancharatnam-Berry phase, the selected width and length are 118 nm and 191 nm, respectively, yielding a transmission coefficient of 0.96. For the isotropic metasurface units, the selected points are positioned along the diagonal line of the phase map to ensure that no leaked LCP component from the transformation metasurface is converted to the RCP component. A total of 20 gradients are selected, each with a transmission coefficient greater than 0.85.

The silicon metasurfaces were fabricated on a glass substrate through a series of processes, including silicon deposition, patterning, lift-off, and etching. First, a 600-nm-thick amorphous silicon (a-Si) film was deposited on the substrate via plasma-enhanced chemical vapor deposition (PECVD). A 150-nm-thick polymethyl methacrylate (PMMA) resist layer was then spin-coated, followed by a conductive protective coating to prevent charge accumulation. After patterning by standard electron beam lithography and development, a 20-nm-thick chromium layer was deposited through electron beam evaporation. The predefined structures were transferred to silicon pillars through inductively coupled plasma reactive ion etching using the chromium mask after a lift-off process. Finally, the residual chromium mask was removed using a standard wet etching process.

**Supplementary Note 6:** **Optical setup for the experimental implementation**


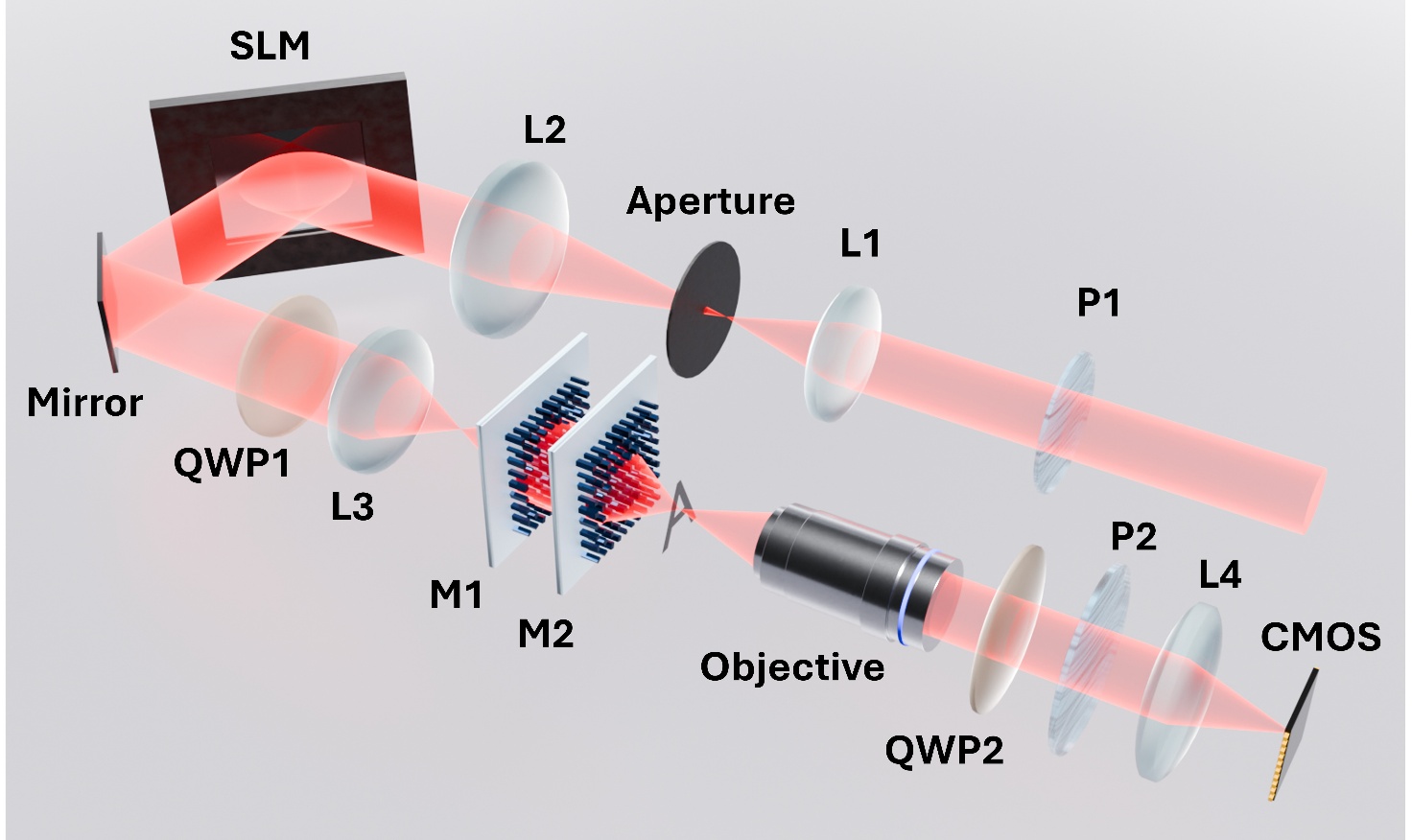


**Figure S7. Optical setup for the experimental implementation.** P: polarizer. L: lens. SLM: spatial light modulator. QWP: quarter-wave plate. M: metasurface. CMOS: Complementary metal-oxide-semiconductor.

The incident beam is generated from an ultra-high-power supercontinuum fiber laser (Fianium WhiteLase SC480). The focal lengths of L1 and L2 are 100 mm and 200 mm, respectively, forming a telescope system to expand the beam size. P1 and QWP1 are combined to generate LCP light. The OAM generation is performed using a phase-only spatial light modulator (Holoeye LETO-3) with a resolution of 1920 × 1080 and a pixel size of 6.4 μm. An aperture is placed between L1 and L2 to adjust the incident beam size on the transformation metasurface (M1). L3, with a focal length of 100 mm, focuses the aligned beam onto the metasurface. A 10× objective lens (NA = 0.3) is used for imaging the focal plane. P2 and QWP2 are combined to filter the RCP light. The intensity distribution is recorded using a CMOS camera (IDS U3-3680XCP-M-NO) with a resolution of 2592 × 1944 and a pixel size of 2.2 μm, after being focused by a lens L4 with a focal length of 100 mm. To be noticed, the incident beam on the transformation metasurface is not at the waist of the Gaussian beam, which introduces an extra phase distribution. This phase is compensated for by loading a lens function with a focal length of 4 mm on the transformation metasurface.

**Supplementary Note 7: The additional analysis for the linear OAM sorter and the simulation results for the two-dimensional OAM sorter**


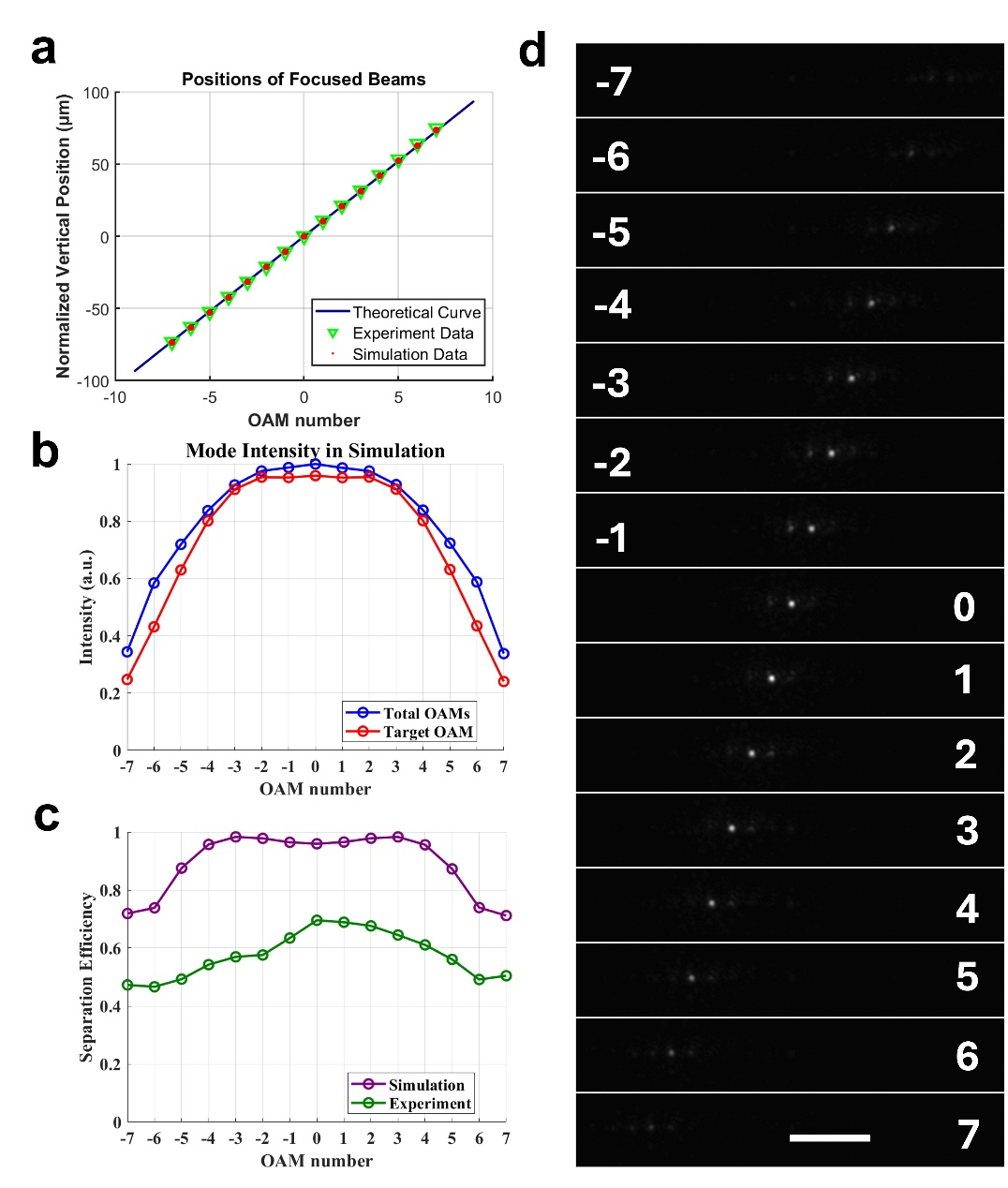


**Figure S8. The experiment results of two-dimensional ultra-compact OAM sorters. a**) The central positions of the focused beams correspond to the input OAMs. **b**) The mode intensity for target OAM and total OAMs in simulation. **c**) The separation efficiency in the simulation and experiment. **d**) The intensity distribution on the focal plane for OAMs from -7 to 7 in the experiment with the diameter of the incident beam is 118 μm. Scale bar: 20 μm.

In both the simulation and experiment, the interval between two adjacent modes is determined by the phase gradients on the compensation metasurface and the focal length, which is calculated to be 10.4 μm. The simulated and experimental average full width at half maximum (FWHM) of the output beams are 2.36 μm and 2.4 μm, respectively.

The separation efficiency for a specific mode is defined as:

$$\text{Separation Efficiency}=\frac{I_{i}}{\sum_{j} I_{j}},$$

Where *I_i_* represents the intensity of the *i*^th^ mode. In the data analysis process, *I_i_* is calculated by integrating the total intensity within a circular region with a diameter of 5 μm, centered at the output beams. The corresponding inter-mode crosstalk is defined as:

$$\text{Crosstalk (dB)}=10\log_{10}\left(\frac{\sum_{i\neq j} I_{i}}{\sum_{j} I_{j}}\right).$$

For OAM values ranging from -5 to 5, the simulated and experimental inter-mode crosstalks are -13.19 dB and -3.96 dB, respectively.


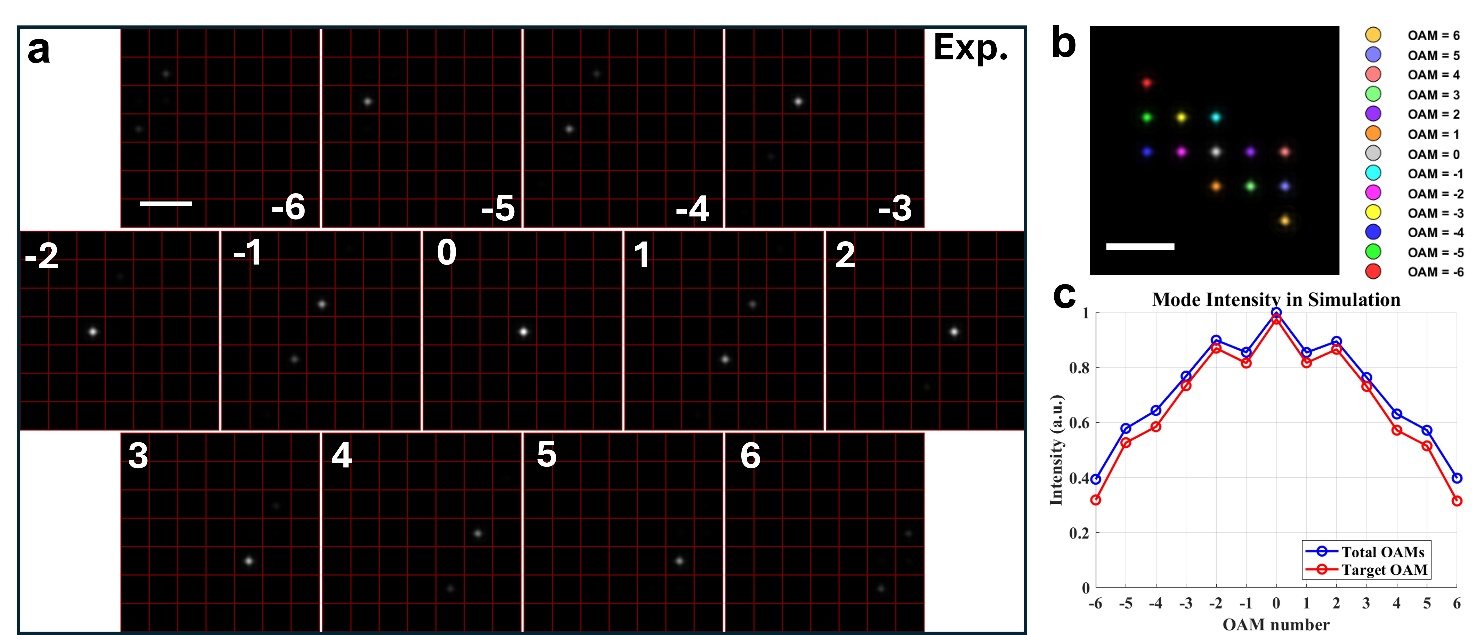


**Figure S9. The simulated results of two-dimensional ultra-compact OAM Sorters. a**) The intensity distribution on the focal plane for OAMs from -6 to 6 in the simulation. **b**) the superposition of all OAM modes in (a). **c**) The mode intensity for target OAM and total OAMs in the experiment. The scale bar in (a) and (b) is 20 μm.

During the optimization of the two-dimensional OAM sorter, minor mode impurity occurs when OAM = ±1 (Figure S9a). However, these impurities do not affect other channels since there is no overlap. For OAM values ranging from -5 to 5, the simulated and experimental inter-mode crosstalks are -14.20 dB and -5.15 dB, respectively.


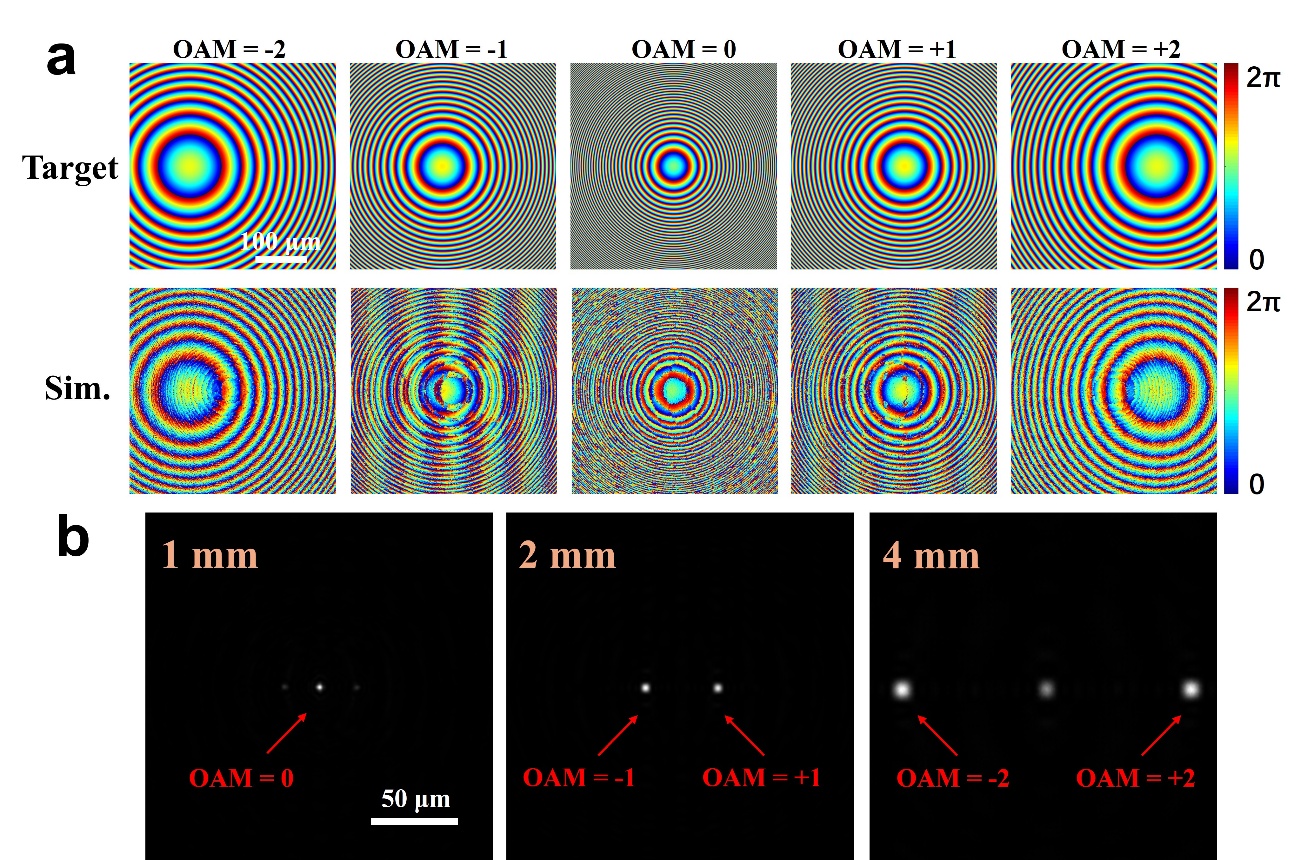


**Figure S10.** A three-dimensional OAM sorter with five channels. **a**) The target and optimized phases for 5 different OAM channels. Each channel are loaded with a bonus focal wavefront. The focal length for OAM = 0, ±1 and ±2 are 1 mm, 2 mm and 4 mm, respectively. **b**) The intensity distribution on the three focal planes.

Three-dimensional OAM sorting can be realized within our design framework. As an example, we demonstrate a 5-channel OAM sorter, in which a distinct focal wavefront is assigned to each channel, allowing all OAM channels to be simultaneously focused on different three-dimensional positions in space. As shown in Figure R4, when illuminated with OAM = 1, a single bright spot appears on the focal plane at 1 mm. The same holds for other OAM modes, where only the corresponding OAM channel exhibits the brightest focused spot on its respective focal plane.

**Supplementary Note 8: The design of a 4-channel holography based on the OAM-selective strategy**


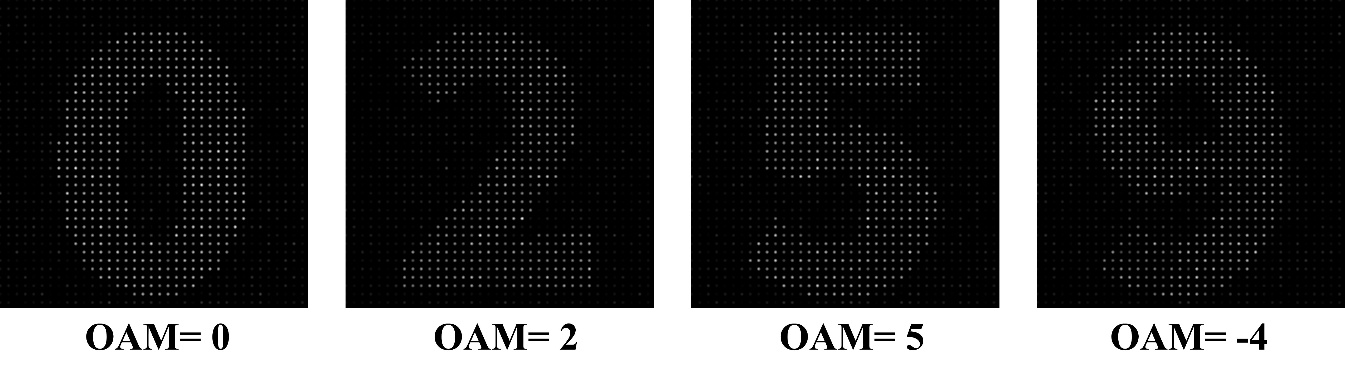


**Figure S11. The original four holographic images designed by the Gerchberg-Saxton algorithm.** Four images are designed separately with encoded OAM = 0, 2, 5, and -4. The image size is 400 μm × 400 μm.

To achieve a 4-channel holography based on the OAM-selective strategy, the four target images must be designed independently. Here, the diameter of the incident Gaussian beam is set to 370 μm, matching the value used in the non-injective optimization. The focal length for the optical Fourier transformation is set to 1.5 mm, corresponding to the distance between the transformation metasurface and the focal plane in the non-injective optimization. Thus, the diameter of the zero-order beam on the focal plane is 4.05 μm. The sampling interval is chosen as 12 μm.

The multiplexed and loaded OAMs are 0, 2, 5, and -4, with the largest OAM difference ΔOAM = 9. Following the OAM-selective strategy, the complex fields of these four holographic plates are added together, whose phase distribution is selected as the final design. The diameter of the aperture array filter is 6 μm. The energy efficiency is defined as the ratio of the integrated intensity at the target image points (after applying the filter) to the total intensity before the filter is applied.

**Supplementary Note 9: The simulation results for angle multiplexing and amplitude multiplexing**


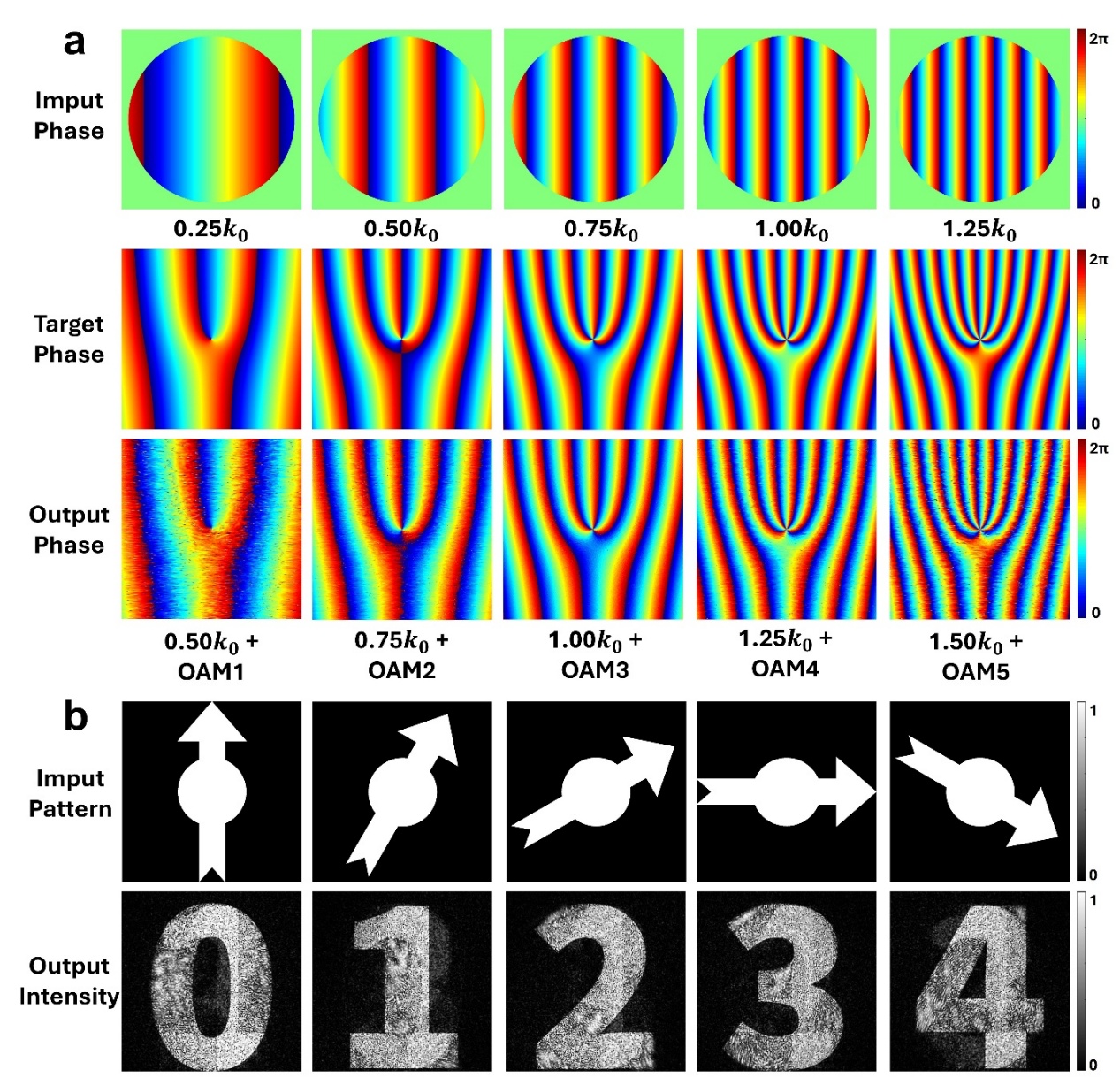


**Figure S12. The simulated results for angle multiplexing and amplitude multiplexing. a**) The wavefront multiplexing from different transverse momentum to a combination of transverse momentum and OAMs. **b**) The wavefront multiplexing from different clock-shaped amplitude to patterns with corresponding numbers. Image size: 400 μm.

The non-injective optimization is capable of diverse wavefront multiplexing. Figure S12a demonstrates an angle-multiplexed function to enable the momentum transformation. The input phase distributions for channel *N* = 1 to 5 are phase gradients with different horizontal wavevectors from 0.25 *k_0_*​ to 1.25 *k_0_*​ with a beam diameter of 370 μm, where *k_0_*​ = 1/(12 μm). The target wavefronts are set as the overlap between increased horizontal wavevectors and different OAMs, expressed as (0.25*N* + 0.25) *k_0_* with an OAM of *N*. The optimized results match well with the design, indicating the ability for the non-injective optimization to map different wavefronts.

Another example demonstrates the potential of amplitude multiplexing for non-injective optimization. The input patterns are clock hand images pointing to different time directions. Although large areas in the center of these input images are superposed, the output patterns still exhibit clear patterns with minimal crosstalk.

**Supplementary Note 10: The estimation for the capacity limit and the computational complexity**


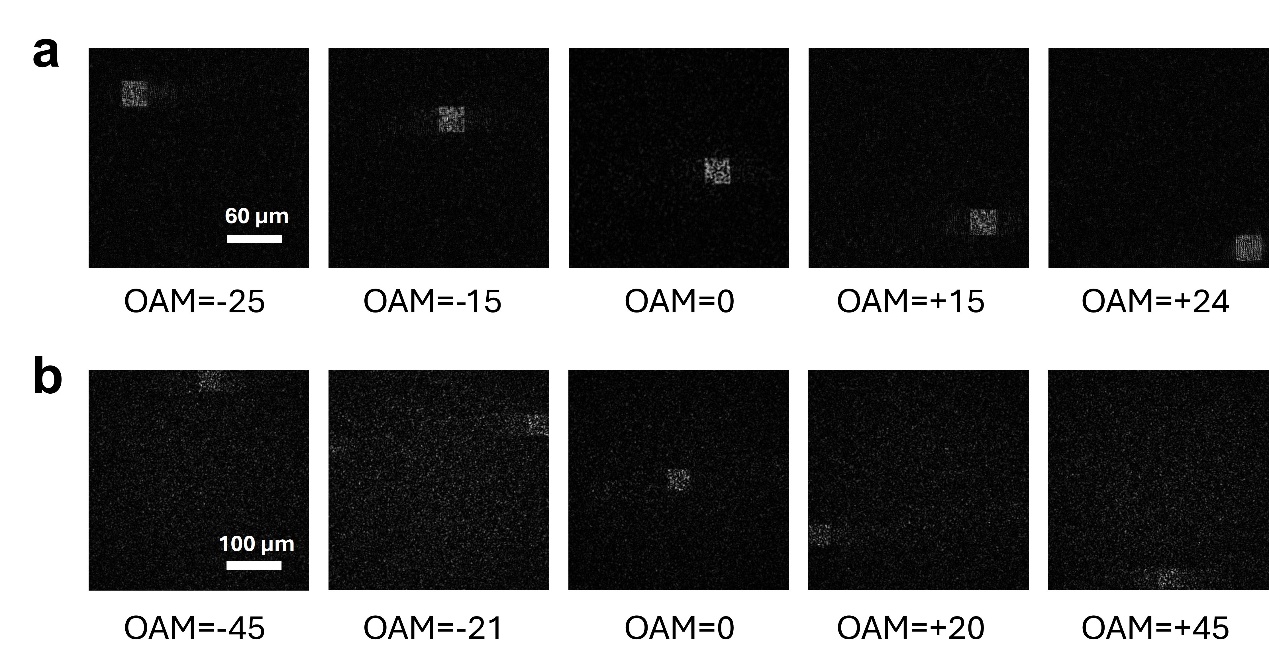


**Figure S13. The multiplexing results with 50 and 100 channels. a**) Image multiplexing with 50 channels corresponding to OAMs from -25 to 24. **b**) Image multiplexing with 100 channels corresponding to OAMs from -50 to 45.

To estimate the maximum capacity of the non-injective optimization, we test two large channel counts: 50 and 100. For 50-channel multiplexing, the target images consist of adjacent 30 μm square blocks, which remain clearly distinguishable in the results (Figure S13a). The average energy efficiency at the target areas is calculated as 15%, corresponding to a signal-to-noise ratio (SNR) of 10.46 dB. To be noticed, if the same capacity is achieved by the OAM-selective strategy, the average energy efficiency is smaller than 2%.

For 100-channel multiplexing, the target images consist of adjacent 40 μm square blocks, with stronger noise observed (Figure S13b). The average energy efficiency is 4.7%, corresponding to an SNR of 6.89 dB. According to the Rose criterion, which requires an SNR of at least 5 to reliably distinguish image features, the multiplexing limit can be identified as 100 channels.

The memory and computational complexity of joint optimization can be evaluated from its mathematical formulation. The main computational step in the algorithm is the angular spectrum propagation, which primarily consists of fast Fourier transform (FFT). Therefore, the time complexity is the same as that of the FFT, namely

$$O \left(N_{x}N_{y}\log(N_{x}N_{y})\right),$$

where *Nx* and *Ny* denote the number of pixels of the metasurfaces along the *x* and *y* directions, respectively. Similar to a convolutional neural network, the computational resource requirement increases linearly with the number of input channels.

In this work, our algorithm does not require extensive computational resources and can be executed on a personal workstation. All simulations were performed on a workstation equipped with an Intel Core i7-10750H CPU (2.60 GHz), 16 GB RAM, and an NVIDIA GeForce RTX 2060 GPU with 6 GB memory. For a 10-channel multiplexing with a simulation area of 2000 × 2000 pixels, each iteration required approximately 2.3 s, and about 200 iterations were sufficient to achieve reliable results. For a 100-channel multiplexing with a simulation area of 1000 × 1000 pixels, each iteration required approximately 6.3 s.

**Supplementary Note 11: The fabrication and alignment error tolerance**

Here, we estimate the error tolerance arising from alignment and fabrication. As an example, we design a two-channel OAM-multiplexed holography system with OAM = 1 and OAM = 2. For convenience in calculating crosstalk, the target images are set to be spatially separated. Figure R11 illustrates the change in the output images induced by lateral displacement. As the transverse displacement increases, the OAM = 1 mode gradually vanishes, while the OAM = 2 mode emerges. This occurs because the alignment displacement shifts the coordinate center of the input OAM, causing the input OAM to be decomposed into other OAM components. Here, we calculate energy loss curves for both the transverse and longitudinal directions. By setting a 50% threshold as the tolerance limit, the lateral displacement tolerance is determined to be approximately 2 μm. Therefore, in the experiment, a high-accuracy translation stage with a resolution of 1 μm is required.


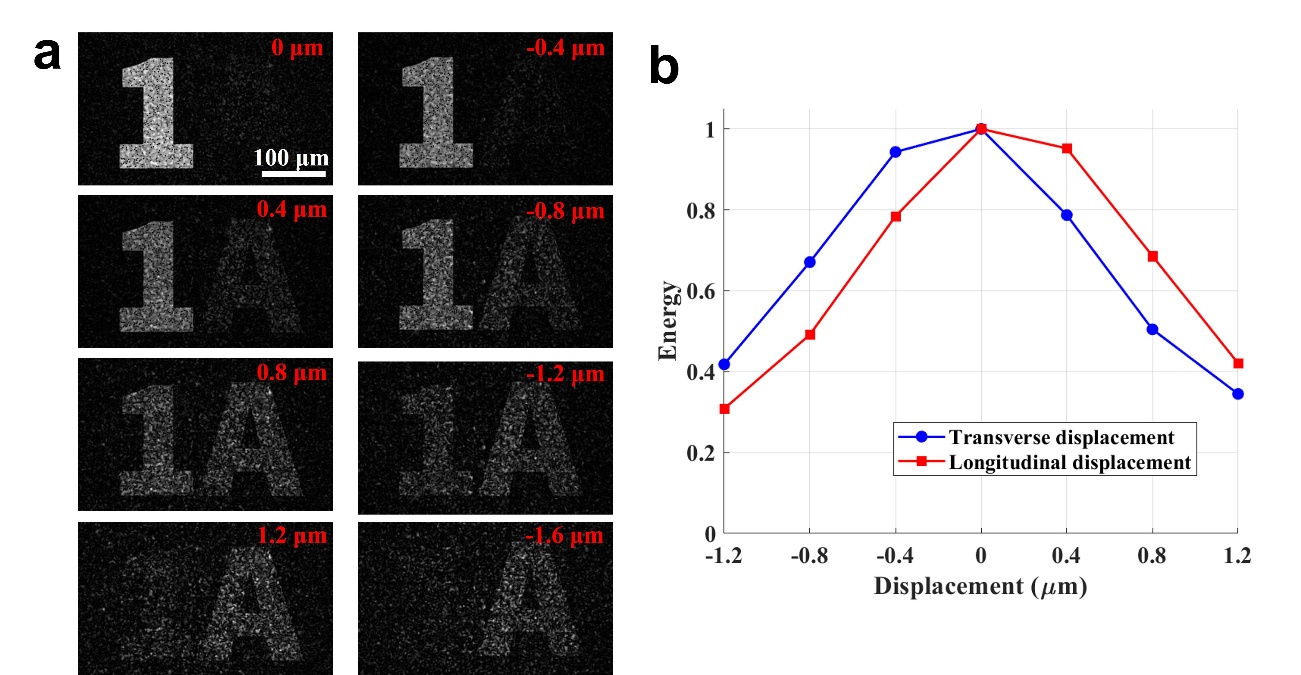


**Figure S14.** The output error of two-channel OAM multiplexing induced by lateral displacement between metasurfaces. **a**) The intensity distributions of imaging plane for different displacements. **b**) The energy loss of the channel with OAM = 1 under different transverse displacements. The left number is encoded with OAM = 1, while the right number is encoded with OAM = 2.

As shown in Figure S15, compared with lateral displacement, the system exhibits better tolerance to both the rotation angle and the distance between the metasurfaces. The calculated tolerance for the rotation angle reaches 1.4°, while the axial displacement tolerance is approximately 12 μm.


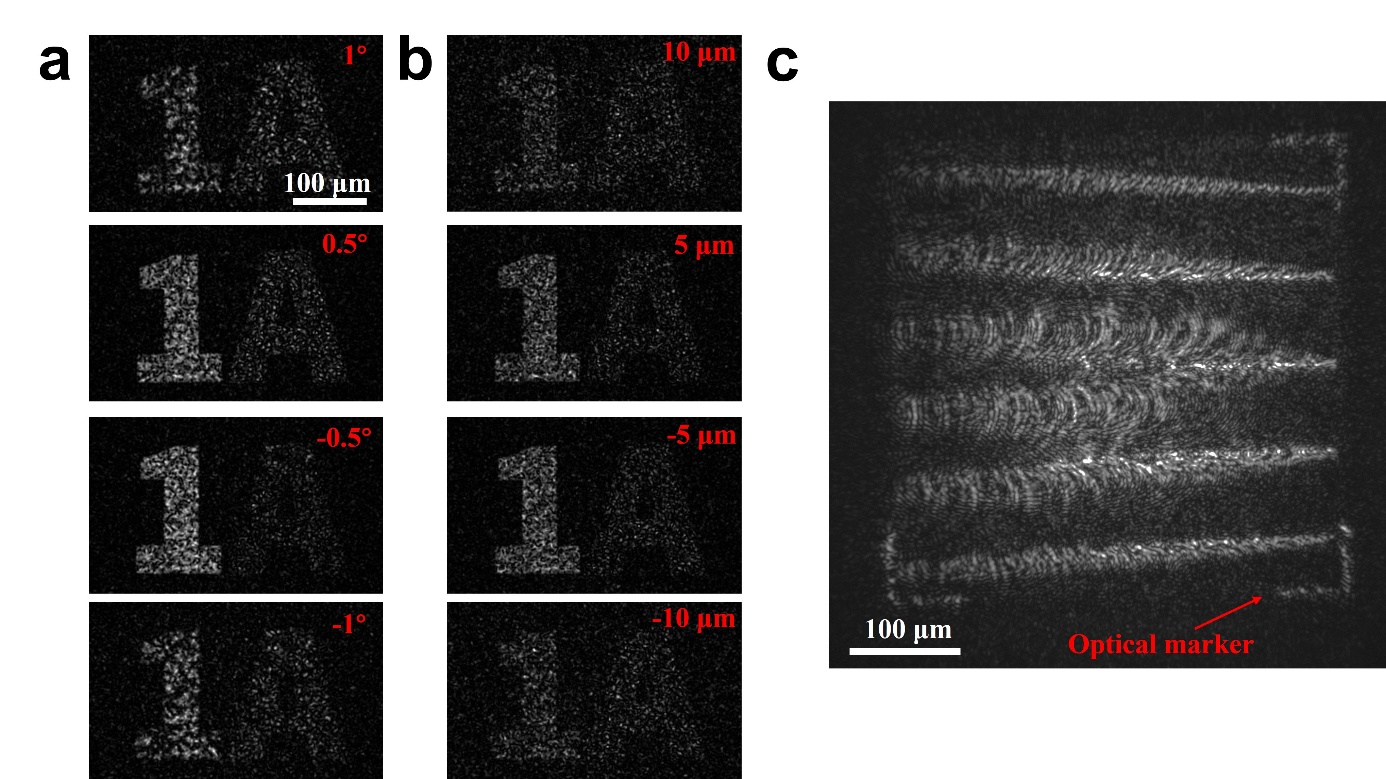


**Figure S15.** The output error of two-channel OAM multiplexing induced by the rotation angle and the distance between two metasurfaces. **a**) The intensity distributions of imaging planes for different rotation angles. **b**) The intensity distributions of imaging plane for different distances. **c**) The optical marker on the compensation plane of the OAM sorter used for experimental alignment.

To better reduce alignment errors, we propose a corner-assisted alignment method. Figure S15c shows an optical corner marker obtained from the experimental images of the compensation plane of an OAM sorter. In the simulation, an additional phase distribution is applied on the transformation plane to generate such an optical corner marker focused onto the compensation plane, with the same size as the compensation metasurface. This allows the compensation metasurface to be conveniently adjusted into the correct square region using a camera.


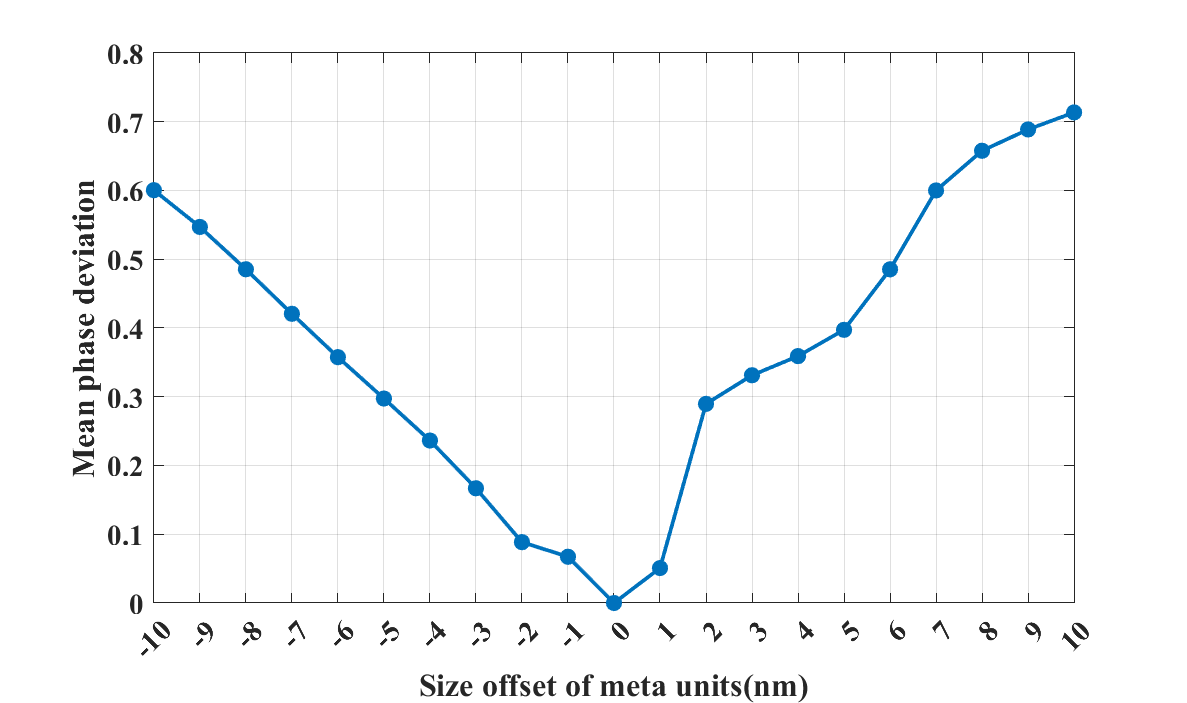


**Figure S16.** The phase deviation curve with respect to the size offset of the meta-units.

Regarding the fabrication process, possible size variations may arise from imperfections in the Cr mask or from the etching procedure. To assess their impact, we evaluate the average phase deviation with respect to the size offset of the employed meta-units. As shown in Figure S16, the selected Si pillars exhibit strong robustness against size offsets. This is because the birefringent units used in the compensation metasurfaces are designed as square pillars. When the pillar size increases along both directions, the phase of each pillar in the library correspondingly increases, thereby reducing the relative phase deviations. Even a size offset of 10 nm introduces only a phase deviation of 0.7 rad.
